# Identification and verification of YBX3 and its regulatory gene HEIH as an oncogenic system: A multidimensional analysis in colon cancer

**DOI:** 10.3389/fimmu.2022.957865

**Published:** 2022-08-18

**Authors:** Yiming Sun, Zhixi Li, Wensheng Wang, Xiuyang Zhang, Wenjing Li, Guangsheng Du, Jiuheng Yin, Weidong Xiao, Hua Yang

**Affiliations:** ^1^ Department of General Surgery, The Second Affiliated Hospital of Army Medical University, Chongqing, China; ^2^ Bengbu Medical University, Bengbu, China; ^3^ Department of Stem Cell and Regenerative Medicine, The Southwest Hospital of Army Medical University, Chongqing, China; ^4^ Department of General Surgery, Chongqing General Hospital, Chongqing, China

**Keywords:** lncRNA-HEIH, YBX3, prognostic and molecular biomarker, immune-cell infiltration, immunotherapy LncRNA-HEIH, immunotherapy

## Abstract

The novel gene YBX3 is important for regulating translation and RNA catabolism and encodes a protein with a highly conserved cold-shock domain. However, its pathogenic roles across cancers (*e*.*g*., colon cancer) and its regulation remain unclear. We identified the pathogenic roles of YBX3 and its regulatory lncRNA HEIH in various cancers and investigated their effects on tumor progression in colon cancer. Methods including RNA pull-down, MS, and TMA of 93 patients, qPCR of 12 patients with diverse clinicopathologic stages, and western blotting were performed. The pancancer analysis showed that YBX3 expression varies significantly among not only cancer types but also molecular and immune subtypes of the same cancer. Furthermore, its expression in colon cancer is clinically significant, and there is an obvious negative regulatory association between HEIH and YBX3. Among various cancers, especially colon cancer, YBX3 is more related than HEIH expression to the clinical features and prognosis of subgroups. The receiver operating characteristic analysis showed that HEIH and YBX3 have similar predictive capacity in various cancers. The analysis of differentially expressed genes in colon cancer revealed that they have similar hub gene networks, indicating an oncogenic system with a strong overlap. The results also suggest that YBX3 is associated with tumor immune evasion *via* different mechanisms involving T-cell exclusion in different cancer types and by the tumor infiltration of immune cells. Interestingly, scRNA-seq revealed that HEIH inhibits this phenomenon. Our results also suggest that YBX3 expression is associated with immune or chemotherapeutic outcomes in various cancers, and YBX3 exhibited a higher predictive power than two of seven standardized biomarkers for response outcomes and overall survival of immune checkpoint blockade subcohorts. In colon cancer cell lines, lncRNA-HEIH and YBX3 associate. MS confirmed that YBX3 was pulled down with HEIH, and western blot showed that HEIH knockdown disinhibited YBX3. This study strongly suggests that lncRNA-HEIH/YBX3 is a pancancer immune-oncogenic system and could serve as a biomarker for diagnosis and prognosis and as a therapeutic target, especially in colon cancer.

## 1. Introduction

Long-noncoding RNAs (lncRNAs) are RNA transcripts that are more than 200 nucleotides in length but have little protein-coding potential (1). lncRNAs act in a wide range of cellular and molecular processes, including chromatin remodeling, gene regulation, and cell proliferation, and can bind or regulate diverse biomolecules, including miRNAs, DNAs, or proteins to exert their biological functions (2–4). Among numerous interactions and regulatory relationships, lncRNAs primarily perform their functions by interacting with other proteins, and they also have been shown to serve as guides, signals, decoys, and scaffolds for various proteins (5, 6) to regulate the location, stability, and activity of the proteins and can participate in the protein-mediated modulation of the immune response. Accumulating evidence indicates that interactions between lncRNAs and their corresponding proteins play a critical role in human diseases and may provide insights for therapies (7).

A class of RNA-binding proteins (RBPs), the YBX family, is comprised of transcription factors mainly expressed on epithelial cells that play an important role in various biological processes, including epithelial cellular development, differentiation, stress, and organogenesis (8). YBX3, also called CSDA or DBPA, is the most recently identified member of this family. Some studies have found that YBX3 can promote the proliferation of breast cancer cells, inhibit the proliferation of kidney and gastric cancer cells, and modulate the function of tight junction (TJ) proteins in bladder cancer (9, 10). Similarly, the long noncoding RNA HEIH (lncRNA-HEIH) is a recently discovered lncRNA involved in regulating a variety of malignant phenotypes and drug resistance, such as promoting HCC cell proliferation, invasiveness, and migration capability *via* the EMT pathway and deregulating classical microRNAs to promote the progression of breast and gastric cancer (11–14). However, the expression, biological function, and diagnostic and prognostic values of these two factors in other cancers remain unclear. Both YBX3 and lncRNA HEIH regulate the therapeutic response through certain common molecular mechanisms, but the role of the tumor regulation system composed of lncRNA-HEIH/YBX3 in the tumor microenvironment (TME) of diverse tumors, especially in colon cancer, has not been fully investigated. Hence, in this study, we explored the effect of the RBP YBX3 on various genes involved in multiple hallmarks of cancer and the effect of its upstream lncRNA HEIH on oncogenesis and progression across cancers, particularly their role in colon cancer. Furthermore, we performed experiments including RNA pull-down assays, mass spectroscopy, analyses of a TMA constructed from surgical specimens, qPCR analysis of specimens of different clinicopathological stages, and western blot to evaluate YBX3 protein expression in HEIH-knockdown cell lines to comprehensively evaluate lncRNA-HEIH/YBX3 as a biomarker for diagnosis and prognosis across cancers and as a promising molecular target for colon adenocarcinoma (COAD).

## 2. Materials and methods

### 2.1 Comprehensive bioinformatics analysis of HEIH/YBX3 across TCGA cancer types

#### 2.1.1 YBX3 and HEIH expression analysis in pan-cancer

Clinical information and RNA-sequencing (RNA-seq) data were acquired from The Cancer Genome Atlas (TCGA) data across 33 types of tumors, and standardized mRNA data of 17,832 samples obtained from normal tissues were acquired from the Gene Expression Omnibus (GEO) genotype–tissue expression database *via* UCSC XENA. The tumor cell line data used for pancancer analysis was acquired from the Cancer Cell Line Encyclopedia. The statistical data analysis was performed using R (version 3.6.3), and the results were visualized by the ggplot2 package. The Wilcoxon rank sum test was applied to compare the different statistics, and the standards of the statistical tests were set as follows: *P* ≥ 0.05, ns (non-significant); *p < 0.05; **p < 0.01; ***p < 0.001.

#### 2.1.2 YBX3 expression among various molecular and immune subtypes across cancers

TISIDB, a database for investigating tumor–immune interactions, was applied to study the relationships between YBX3 expression and immune subtypes and molecular subtypes across cancers.

#### 2.1.3 Evaluation of the diagnostic value of HEIH/YBX3

A receiver operating characteristic (ROC) curve was applied to evaluate the diagnostic value of HEIH/YBX3 across cancers. The range of ROC–area under the curve (AUC) indicates the accuracy and diagnostic performance of HEIH/YBX3. Moreover, the higher the AUC value is, the better the diagnostic efficacy. The specific classifications of AUC are listed as follows: an AUC of 0.5–0.7 indicates relatively low accuracy, an AUC of 0.7–0.9 indicates medium accuracy, and AUC >0.9 indicates high accuracy.

#### 2.1.4 Survival analysis of HEIH/YBX3

The relationship between HEIH/YBX3 expression and pancancer survival, including overall survival (OS), disease-specific survival (DSS), and progression-free interval (PFI) was analyzed using Kaplan–Meier plots. To achieve a more macroscopic evaluation of certain tumors, such as glioma (composed of LGG and GBM) and lung cancer (composed of LUSC and LUAD), some TCGA data were selectively combined before analysis. In particular, the expression of HEIH and YBX3 and the corresponding tumor prognosis among digestive tract cancers were further analyzed. The R survminer package was used to plot the survival curves, and the survival probabilities were determined by the R survival package. Moreover, the hypothesis test was evaluated by using Cox regression with a cutoff value of 0.05.

#### 2.1.5 Associations between HEIH/YBX3 expression and diverse clinical features in COAD

In COAD, violin plots were generated by the R ggplot2 package to present the HEIH/YBX3 expression levels of patients with different clinical features in COAD. In this part, all calculations using fragments per kilobase per million (FPKM), the RNA- seq data format in the TCGA database, were performed on log2(FPKM+1)-transformed expression values. The association between HEIH/YBX3 expression and the clinical characteristics in COAD was strong. Therefore, the similarity of the extent of their influence on the clinical features of COAD was further investigated. The Wilcoxon rank sum test was considered for statistical analysis with a cutoff value of 0.05 (*P < 0.05; **P < 0.01; ***P < 0.001).

#### 2.1.6 Variable cox regression analysis of HEIH and YBX3 in COAD

To identify the prognostic values of HEIH and YBX3 expression for COAD in terms of survival, including OS, DSS, and PFI, univariate and multivariate analyses were performed using univariate and multivariate Cox regression models, respectively. The relative statistical analysis was performed using the R survival package.

#### 2.1.7 HEIH/YBX3 gene co-expression analysis in COAD

The top 50 co-expressed genes positively and negatively correlated with CLDN6 expression in UCEC were identified. Co-expression information for the top 50 genes with the highest correlation with the HEIH and YBX3 genes was obtained from the COAD-related datasets in the TCGA database. A heat map was generated using the packages heatmap2 and stat. The association between HEIH and YBX3 expression and the expression of the top 10 genes (including five positively correlated genes and five negatively correlated genes) in COAD was visualized in the form of scatter plots utilizing Pearson’s correlation coefficient.

#### 2.1.8 DEGs between different-HEIH/YBX3-expression-level groups in COAD

The different-HEIH/YBX3-expression-level groups of differentially expressed genes (DEGs) were defined as follows: [0, 0.5] means low expression and [0.5, 1] means high expression in COAD utilizing R deseq2 package. The volcano plot was created by the Enhanced Volcano R package with settings of |log(FC)| >1 and adjusted *P*-value <0.05. Then, the ggplot2 package and clusterProfiler package were used to visualize and count the Gene Ontology (GO) and Kyoto Encyclopedia of Genes and Genomes (KEGG) enrichment analyses of DEGs separately in the form of chordal graphs. In addition, a PPI network of DEGs was constructed according to |log(FC)| >2 with the help of STRING, and the hub genes were visualized by CytoHubba in Cytoscape (version 3.7.2) and analyzed by the MCC algorithm.

#### 2.1.9 YBX3 variants, localization, and expression profiles under physiological conditions

The intracellular membrane localization of YBX3 was revealed by the protein topology determined *via* the Protter tool. To clarify the distribution and localization of the YBX3 protein, an indirect fluorescent antibody test was performed in PC-3, MCF-7, and U-2 cells with the help of Human Protein Alas. Meanwhile, the relative expression of YBX3 among diverse tissues was also presented by the GTEx database. Next, the network of functional gene partners of YBX3 and its associated disease network were presented with the help of the Open Targets Platform.

#### 2.1.10 YBX3 expression, relative immune cell infiltration, and ICB analysis

The R cyclize package was used to visualize the correlation between YBX3 expression and infiltration of six diverse types of immune cells in the form of a loop heat map. Furthermore, the correlation of YBX3 expression with three different immunosuppressive cells, which have a likelihood of promoting T-cell exclusion, was also analyzed among all TCGA cancer types. Next, with the aid of the Tumour Immune Dysfunction and Exclusion (TIDE) database, the biomarker relevance of YBX3 compared to standardized cancer immune evasion biomarkers in immune checkpoint blockade (ICB) subcohorts was further evaluated. Then, to explore the relationship between the expression of YBX3 and HEIH and their expression profiles in immune cell infiltration, scRNA-seq data from diverse GSE chips, such as 146771, 16394, and 13955, were visualized by subgroup classification and annotation.

#### 2.1.11 Analysis of the association between therapeutic response and YBX3 expression

The relationship between various clinical chemotherapeutic activities and YBX3 expression was further investigated in diverse databases. Specifically, the effect of YBX3 expression on the clinical response to chemotherapy in breast, glioma, ovary, and colon cancer cohorts across various cancer centers was evaluated with ROC plotter. Furthermore, the correlation between YBX3 expression and cytotoxic T cell (CTL) levels in cancer cohorts among different cancer centers was visualized by the TIDE database in the form of Kaplan–Meier survival curves, which served to indicate ICB, also known as immunotherapeutic response, between cancer cohorts with high and low YBX3 expression.

### 2.2 Experimental verification of the relationship and function of HEIH/YBX3 in colon cancer

#### 2.2.1 Clinical specimen and cell line preparation

The pathology specimens were acquired from the biospecimen repository of Xinqiao Hospital. The 93 patients from our Department of General Surgery, Xinqiao Hospital, all underwent laparoscopic left (or right) hemicolectomy during 2015–2016. All patients signed an informed consent upon admission to our hospital. The application for surgical specimens was approved by the Clinical Ethics Committee of the Second Affiliated Hospital of Army Medical University of the PLA (no. 2022-036-01). The study methodologies strictly conformed to the standards set by the Declaration of Helsinki.

Colon cancer cell lines, including SW620 and HCT116, were purchased from Procell Life Science & Technology Co., Ltd. Then, they were cultured in the corresponding complete medium, which was Leibovitz’s L-15 (PM151010)+10% FBS (164210-500)+1% P/S (PB180120) or MEM+10% FBS (164210-500)+1% P/S (PB180120), in an incubator of 37°C constant temperature with 5% CO_2_.

#### 2.2.2 RNA pull-down and MS

A biotinylated-lncRNA HEIH probe and its antisense RNA probe were synthesized and incubated with cellular protein extract to produce RNA–protein complexes. The RNA–protein complexes were separated from other components in the incubated solution through binding to magnetic beads. After elution, the proteins bound to lncRNA HEIH were detected by MS for further identification.

The RNA pull-down eluate mentioned above was dissolved in 0.1% formic acid with 2% acetonitrile, and then the supernatant was taken into a mass spectrometer (Thermo Scientific Q Exactive, US) with the following parameters: resolution, 70,000; AGC target: 3e6; maximum IT: 40 ms; and scan range: 350 to 1,800 m/z. Finally, MM File Conversion software was used to convert the raw data into the readable MGF format to allow the use of Mascot for sequence alignment on the UniProt website.

#### 2.2.3 TMA construction and IHC

Wax-embedded tissue acquired from our specimen repository was arranged in the form of a microarray after the tissue core was established. Then, the colon adenocarcinoma and its adjacent normal tissue, which refers to the tissues located at least 5 cm from the carcinomas, were all arranged in 0.2-mm intervals. After the tissue microarray (TMA) was completely assembled, the block was sliced into 50 pieces.

After placing the above-mentioned TMA in an oven to evaporate the liquid, the TMA was incubated with primary antibodies (Thermo Fisher Invitrogen ZONAB Polyclonal Antibody, PA5-57080, 1:1,000), followed by incubation with secondary antibodies. Finally, the TMA was applied with DAB substrate for color development and observed under a microscope.

#### 2.2.4 RNA extraction from formalin-fixed tissue and qPCR analysis of HEIH/YBX3 expression

Twelve specimens, which are described above, of colon cancers at different clinicopathological stages (three specimens for each stage) collected at our hospital were randomly selected. After that, RNeasy FFPE Kit (QIAGEN, catalog no. 73504) was used to extract the total RNA for reverse transcription. Next, the YBX3 and HEIH primers (**Supplementary File 1**) were added to the reaction system for qPCR to determine the relative mRNA expression level of HEIH/YBX3 in colon cancer.

#### 2.2.5 Preparation of HEIH siRNA and transfection

The small interfering RNA (siRNA) was designed and synthesized as per HEIH gene cDNA in GenBank. The upstream and downstream segments of HEIH are listed as follows: the PCR products were cloned into the pcDNA3.1 directional expression vector from Invitrogen (USA). Then, target fragments were purified and ligated into a digested pET32a plasmid, and the digested product was directly transformed into *E. coli* DH5-α competent cells. After that, extraction and detection of recombinant plasmid DNA were performed. Finally, the SW620 cell line was transfected with the pcDNA3.1-HEIH plasmid.

#### 2.2.6 WB analysis of the regulatory effect of lncRNA HEIH on YBX3 expression in colon cancer

At 48 h after plasmid transfection, the cells were collected and lysed to extract total cellular protein, and HEIH knockdown efficiency was validated by qPCR. Then, western blot (WB) was performed according to standard procedures with YBX3 primary antibody (ORIGENE, YBX3 rabbit polyclonal antibody, catalog no. TA324558, 1:1,000).

## 3. Results

### 3.1 YBX3 and HEIH expression across cancers

**Figure 1:**
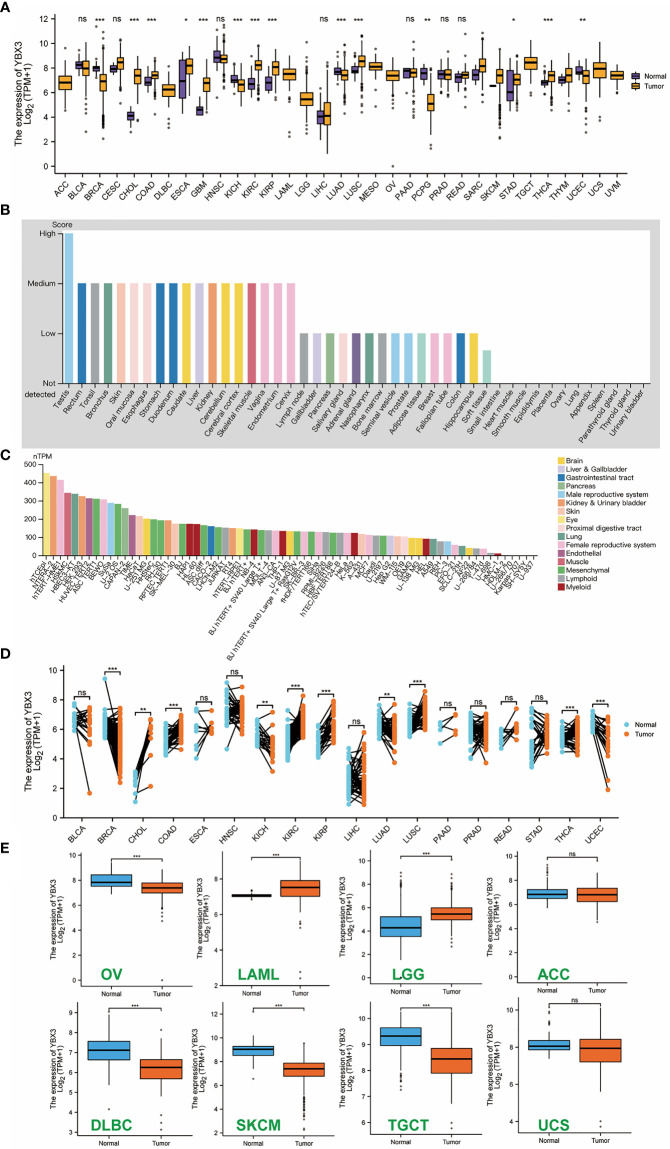
Expression level of YBX3 gene in diverse tumors, normal tissues, and cell lines (*p < 0.05, **p < 0.01, ***p < 0.001). **(A)** YBX3 expression profile in unpaired samples among pan-cancer. **(B)** YBX3 expression in different normal tissues. **(C)** YBX3 expression in diverse cell lines of different organs. **(D)** YBX3 expression in The Cancer Genome Atlas tumors and corresponding adjacent normal tissues in paired samples. **(E)** YBX3 expression in some cancers using data from GTEx databases as controls. ns, non-significant.

A total of 15,776 samples across 33 cancer types in TCGA and the GTEx database were analyzed for YBX3 and HEIH expression. The results (**Figure 1A**) showed that YBX3 was highly expressed across most cancer tissues compared with paracancerous tissues, and the expression level was relatively higher among CHOL (cholangiocarcinoma), COAD, KIRC (kidney renal clear cell carcinoma), KIRP (kidney renal papillary cell carcinoma), LUSC (lung squamous cell carcinoma), and THCA (thyroid carcinoma). Interestingly, the trend of HEIH gene expression was opposite to the trend of YBX3 gene expression among the above-mentioned cancers. The YBX3 expression in different organs and cell lines is presented in **Figures 1B, C**, respectively. In addition, 10,534 samples from the XENA-TCGA database were examined to explore YBX3 expression across cancers in paired samples (**Figure 1D**). For normal tissue data that correspond to several types of tumors in the TCGA database, such as ACC (adrenocortical carcinoma), DLBC (lymphoid neoplasm, diffuse large B-cell lymphoma), LGG (brain, lower grade glioma), OV (ovarian serous cystadenocarcinoma), SKCM (skin, cutaneous melanoma), TGCT (testicular germ cell tumors), and UCS (uterine carcinosarcoma), data from the GTEx database were naturally combined to perform further analysis, and it was found that YBX3 expression was higher in tumors such as OV, DLBC, SKCM, and TGCT than in their corresponding paracancerous normal tissue (*P*-value <0.001). However, the opposite trend was observed in other tumors, including LGG and LAML (also with *P*-value <0.001) (**Figure 1E**).

### 3.2 The relationship between YBX3 expression and immune and molecular subtypes among human cancers

The TISIDB database was used to analyze the role of YBX3 expression in various immune and molecular subtypes of human cancers. The results showed that YBX3 was differentially expressed in immune subtypes of cancers including BLCA (bladder urothelial carcinoma), CESC (cervical squamous cell carcinoma and endocervical adenocarcinoma), COAD, KIRC, KIRP, LGG, LIHC, LUAD, LUSC, PCPG (pheochromocytoma and paraganglioma), and PRAD (prostate adenocarcinoma), and the differences between the subgroups of each tumor mentioned above were also statistically significant, with *P*-value <0.05 (**Supplementary Figure S1–1A**). For BLCA and KIRC, YBX3 was expressed at the highest levels in the TGF-b dominant immune subtype, while for KIRP, LIHC, LUAD, LUSC, and PRAD, YBX3 expression was highest in the wound healing subtype. The overall profile of YBX3 expression across all TCGA cancer types is presented in **Supplementary Figure S1–1B**.

YBX3 gene expression in different molecular subtypes of TCGA cancers is shown in **Supplementary Figure S1–1C** and **Supplementary Figure S1–1D**. The YBX3 expression level presented relatively more variance among molecular subtypes. However, the most evident trend was seen in BRCA, in which the basal subtype presented a higher YBX3 expression level; a similar trend was observed in LUSC.

### 3.3 Diagnostic value of HEIH/YBX3 in multiple cancer types

To evaluate the potential diagnostic value of HEIH/YBX3 for TCGA cancer types, ROC curve analysis was performed (**Figures S1–2**). The results showed that YBX3 expression presented fairly high accuracy (AUC >0.8 or even >0.9) in predicting 10 cancer types (AUC value in parentheses), namely, KIRC (0.911), LUSC (0.811), TGCT (0.813), SKCM (0.944), LUAD (0.849), UCEC (0.810), GBM (0.916), COAD (0.808), CHOL (0.960), and BRCA (0.911). Meanwhile, the presence of the lncRNA HEIH was able to predict the occurrence of 18 cancer types with considerable accuracy: LUSC (0.944), TCCT (0.968), LUAD (0.820), GBM (0.918), COAD (0.875), CHOL (1.000), BRCA (0.815), LIHC (0.819), ESCA (0.876), STAD (0.801), READ (0.910), PRAD (0.871), THCA (0.803), GBMLGG (0.923), LGG (0.925), OV (0.953), THYM (0.843), and UCS (0.900). Furthermore, it is noteworthy that, among the cancer types mentioned above, YBX3 and HEIH showed a similar predictive accuracy in seven types (nearly 50% had a high accuracy, with AUC >0.9), which accounted for 70% of the cancer types that it could predict. In that sense, YBX3 and HEIH have a similar diagnostic value.

### 3.4 Prognostic value of HEIH/YBX3 in various cancers

The association between YBX3 expression and OS, DSS, and PFI was further analyzed in TCGA cohorts by Cox regression (**Supplementary Figure S1–3A**), and the results revealed that, in the KIRC cohort, a higher YBX3 expression was associated with a worse prognosis in terms of OS (HR = 1.38, 95% CI: 1.02–1.86, *P* = 0.036), DSS (HR = 1.62, 95% CI: 1.10–2.38, *P* = 0.014), and PFI (HR = 1.85, 95% CI: 1.35–2.56, *P* < 0.001). For brain cancer, the cohorts of LGG and GBM were combined, and the results showed a trend similar to that of KIRC in OS (HR = 3.87, 95% CI: 2.95–5.08, *P* < 0.001), DSS (HR =3.91, 95% CI: 2.94–5.20, *P* < 0.001), and PFI (HR = 2.57, 95% CI: 2.06–3.20, *P* < 0.001). The same trend was observed for the PAAD cohort in OS (HR = 1.54, 95% CI: 1.02–2.33, *P* = 0.039), DSS (HR =1.72, 95% CI: 1.08–2.75, *P* = 0.024), and PFI (HR = 1.52, 95% CI: 1.03–2.24, *P* = 0.034).

It is worth mentioning that, for tumors that occur in the upper body, such as ACC, HNSC, and lung cancers (including LUSC and LUAD), a higher YBX3 expression indicates a worse prognosis in terms of OS, DSS, and PFI, all with *P <*0.05 (**Supplementary Figure S1–3B**). Surprisingly, when we explored the correlation between YBX3 and HEIH expression and the outcomes of digestive tract cancers, we found that, in most digestive tract cancers, such as ESAD, STAD, and COAD, the expression of the two genes presented diametrically opposed relationships with prognosis—namely, a higher expression of YBX3 predicted a worse prognosis in digestive tract cancers, while a higher expression of HEIH predicted a better prognosis of the same cancer types (**Supplementary Figures S1–3C, D**).

### 3.5 HEIH/YBX3 expression is correlated with diverse clinical characteristics in colon cancer

Given the above-mentioned results, the effect of HEIH/YBX3 expression on diverse clinical characteristics in COAD was further explored (**Supplementary Figure S1–4**, **Supplementary Tables S1**, **S2**). The results showed that a higher expression of the YBX3 gene indicates primary colon cancer with high lymph node stage (≥1), metastasis status, serum CEA level (≥5 ng/ml), lymphatic invasion, and/or residual tumor after surgery (≥1), all with *P <*0.05. Conversely, it was interesting to note that the HEIH expression level was higher in paracancerous tissue of colon cancer patients than in the corresponding cancer tissue and was also higher in patients with higher nodal stage (≥1) and/or metastasis.

### 3.6 Association between prognosis and HEIH/YBX3 expression in different clinical subgroups of COAD

**Figure 2:**
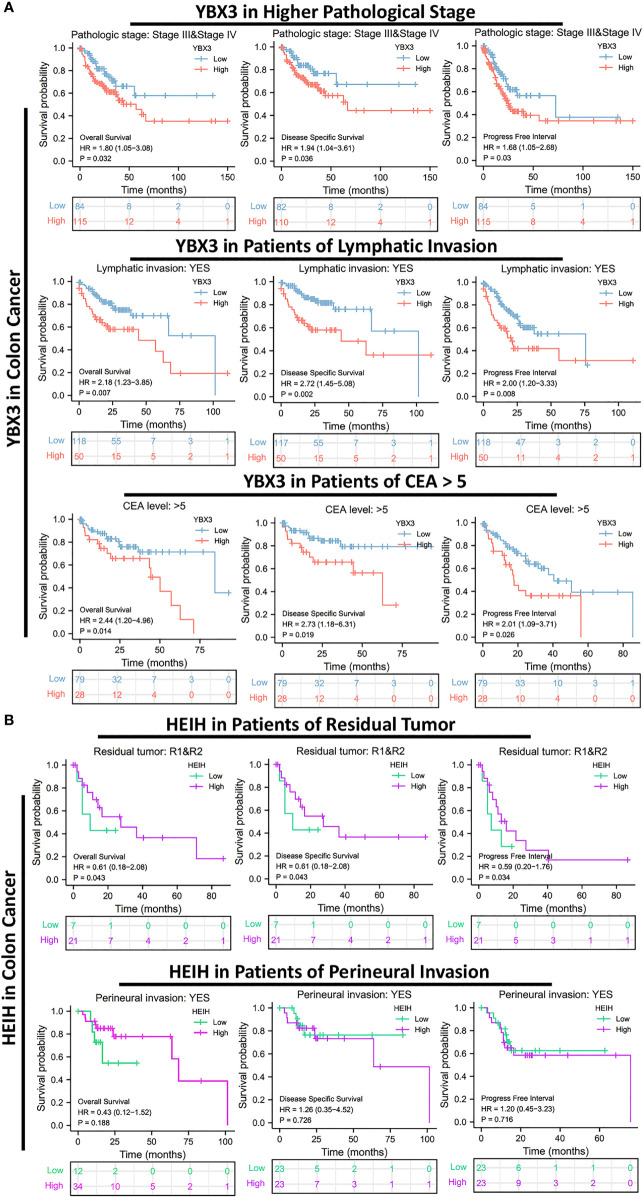
Associations between HEIH/YBX3 expression and the prognosis in different clinical subgroups of colon cancer. **(A)** Effect of YBX3 expression on colon cancer of higher pathological stage, cohorts of lymphatic invasion, and CEA >5. **(B)** Effect of HEIH expression on colon cancer of patients with residual tumor and perineural invasion.

To further investigate the correlations between HEIH/YBX3 expression and prognosis in different clinical subgroups of COAD, the survminer package of R (v3.6.3) was applied to perform a subgroup analysis. The results revealed that, in COAD cohorts, among patients with higher pathological stages (III and IV), a higher YBX3 expression was observed for the subgroups with worse outcomes for OS (HR: 1.8, CI: 1.06–3.08, *P* = 0.032), DSS (HR = 1.94, CI: 1.04–3.61, *P* = 0.036), and PFI (HR: 1.58, CI: 1.05–2.68, *P* = 0.03). For the patients with lymphatic invasion, we found an increased YBX3 gene expression in subgroups with worse prognosis whether for OS (HR = 2.18, CI: 1.23–3.85, *P* = 0.007), DSS (HR = 2.72, CI: 1.45–5.06, *P* = 0.002), or PFI (HR = 2.00, CI: 1.20–3.33, *P* = 0.008). Moreover, the subgroup of patients with elevated serum CEA levels (>5 ng/ml) had a higher YBX3 expression, leading to an unfavorable prognosis in terms of OS (HR = 2.44, CI: 1.20–4.96, *P* = 0.014), DSS (HR = 2.73, CI: 1.18–6.31, *P* = 0.019), and PFI (HR = 2.01, CI: 1.09–3.71, *P* = 0.026) (**Figure 2A**).

Interestingly, for COAD patients with residual tumors (*R* ≥ 1), a higher expression of HEIH implied a better prognosis in terms of OS (HR = 0.61, CI: 0.18–2.08, *P* = 0.043), DSS (HR = 0.61, CI: 0.18–2.08, *P* = 0.043), and PFI (HR = 0.59, CI: 0.20–1.76, *P* = 0.034). Furthermore, COAD patients suffering from perineural invasion followed a similar trend in Cox regression plots, with a *P*-value >0.05 (**Figure 2B**).

### 3.7 Univariate and multivariate Cox regression analyses in COAD

Cox regression was conducted for the univariate and multivariate analyses of HEIH/YBX3 expression and clinical characteristics in COAD. In a cohort composed of 521 samples from TCGA-COAD, including 41 paracancerous tissues and 480 cancer tissues, clinical pathological stage, primary therapy outcome, age, BMI, residual tumor, CEA level, and lymphatic invasion were significantly associated with OS in the univariate Cox regression analyses (**Supplementary Table S3**). In the multivariate Cox regression analysis, residual tumor was significantly correlated with DSS (**Supplementary Table S4**).

### 3.8 Co-expressed genes and DEGs between the high- and low-HEIH/YBX3-expression groups in COAD

**Figure 3:**
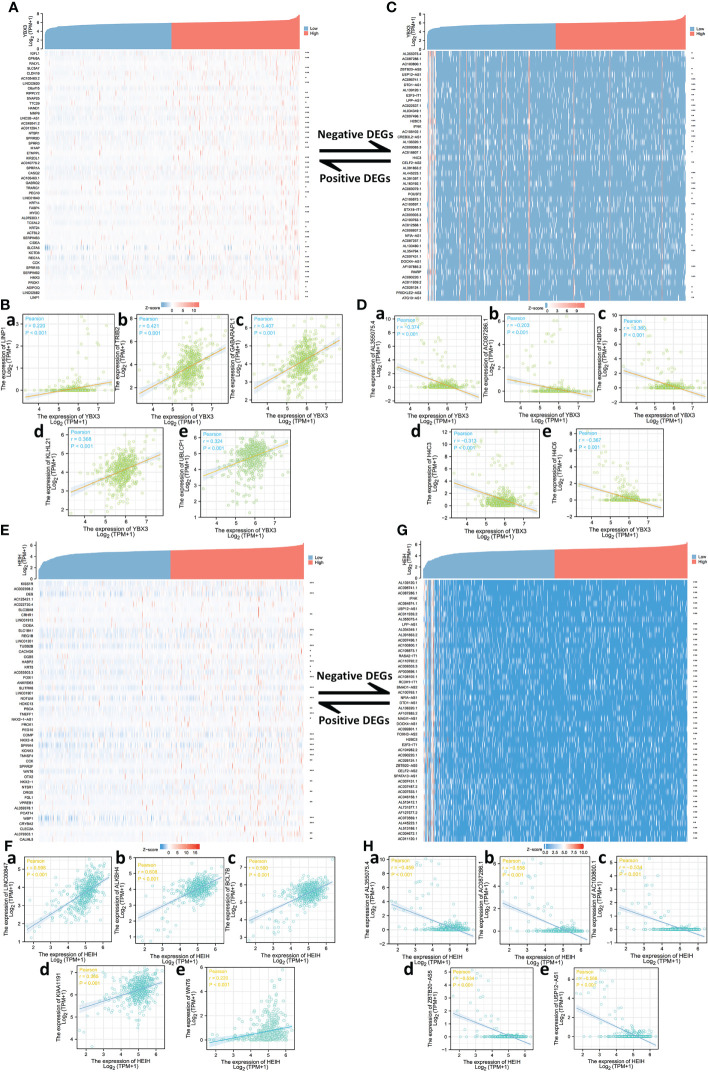
Top 50 genes correlated with YBX3 and HEIH expression in colon adenocarcinoma (COAD). **(A)** Gene co-expression heat map of the top 50 genes positively correlated with YBX3 in COAD. **(B, a–e)** Correlation analysis of the top five genes and YBX3 expression of COAD (positively correlated). **(C)** Gene co-expression heat map of the top 50 genes negatively correlated with YBX3 in COAD. **(D, a–e)** Correlation analysis of the top five genes and YBX3 expression of COAD (negatively correlated). **(E)** Gene co-expression heat map of the top 50 genes positively correlated with HEIH in COAD. **(F, a–e)** Correlation analysis of the top five genes and HEIH expression of COAD (positively correlated). (**G**) Gene co-expression heat map of the top 50 genes negatively correlated with HEIH in COAD. **(H, a–e)** Correlation analysis of the top five genes and HEIH expression of COAD (negatively correlated).

The top 50 correlated genes associated with YBX3 in COAD were explored separately with the help of the STRING and GEPIA2 databases, and the results were visualized in the form of a heat map and scatter diagram by means of the ggplot2 package (v3.3.3, **Figures 3A, B** **)**. The top 10 genes correlated with YBX3 were acquired and included LINP1 (*r* = 0.220), TRIB2 (*r* = 0.421), GABARAPL1 (*r* = 0.407), KLHL21 (*r* = 0.368), and UBLCP1 (*r* = 0.324) as positively correlated genes (**Figure 3Ca–e**) and AL355075.4 (*r* = 0.-374), AC087286.1 (*r* = -0.203), H2BC3 (*r* = -0.360), H4C3 (*r* = -0.313), and H4C6 (*r* = - 0.367) as negatively correlated genes, all with *P*-value <0.001 (**Figure 3Da–e**).

Similarly, the top 50 correlated genes associated with HEIH in COAD were also screened. After using a heat map and scatter plots to visualize the top 50 positively correlated expression genes and the top five genes separately (**Figures 3E, F** **)**, LINC00847 (*r* = 0.598), ALKBH4 (*r* = 0.608), BCL7B (*r* = 0.590), KIAA1191 (*r* = 0.365), and WNT6 (*r* = 0.220) were identified as the most correlated genes (**Figure 3Fa–e**). In **Figure 3G**, we visualized the top 50 negatively correlated expression genes, and the top five are presented in the form of scatter plots in **Figure 3Ha–e**: AL355075.4 (*r* = -0.459), AC087286.1 (*r* = - 0.558), AC100800.1 (*r* = -0.534), ZBTB20-AS5 (*r* = -0.534), and USP12-AS1 (*r* = -0.566), all with *P*-value <0.001.

**Figure 4:**
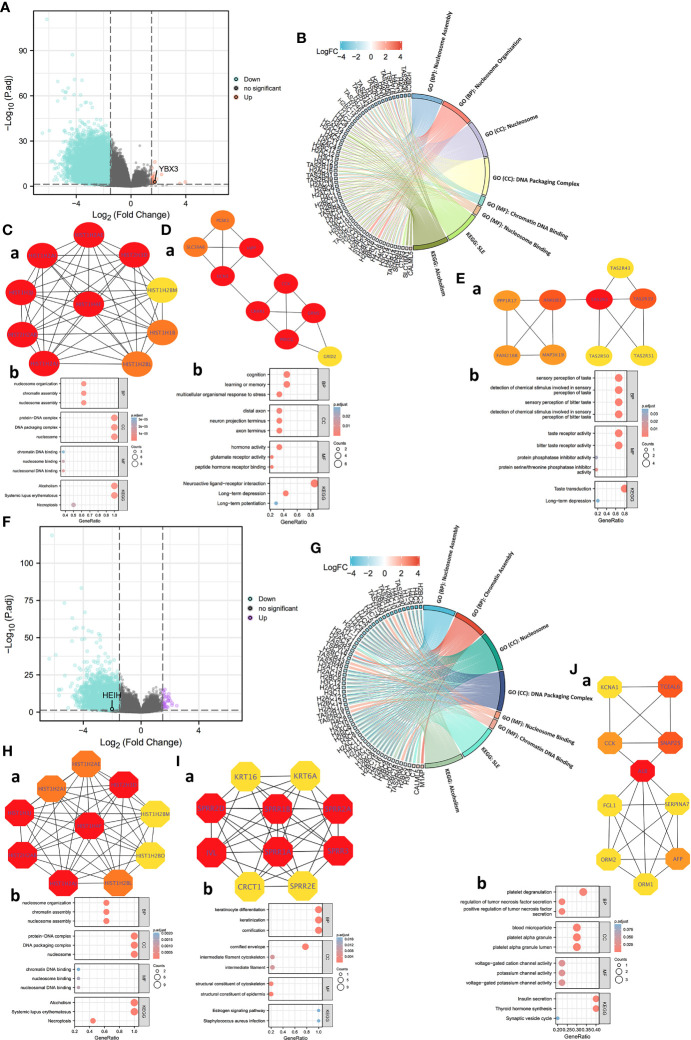
Protein–protein interaction (PPI) network construction and Gene Ontology (GO) and Kyoto Encyclopedia of Genes and Genomes (KEGG) analyses of differentially expressed genes (DEGs) between HEIH/YBX3 high-expression and low-expression groups in COAD. **(A)** Volcano map of DEGs of HEIH expression in COAD (red: upregulation; blue: downregulation). **(B)** GO and KEGG pathway enrichment analyses of DEGs of HEIH in the form of a chordal graph. **(C, a)** Hub genes of PPI network (ranking first) and MCODE2 components identified in the gene lists. **(C, b)** Enrichment analysis of the hub gene network. **(D, a)** Hub genes of PPI network (ranking second) and MCODE2 components identified in the gene lists. **(D, b)** Enrichment analysis of the hub gene network. **(E, a)** Hub genes of PPI network (ranking third) and MCODE2 components identified in the gene lists. **(E, b)** Enrichment analysis of the hub gene network. **(F)** Volcano map of DEGs of YBX3 expression in COAD (purple: upregulation; blue: downregulation). **(G)** GO and KEGG pathway enrichment analyses of DEGs of YBX3 in the form of a chordal graph. **(H, a)** Hub genes of PPI network (ranking first) and MCODE2 components identified in the gene lists. **(H, b)** Enrichment analysis of the hub gene network. **(I, a)** Hub genes of PPI network (ranking second) and MCODE2 components identified in the gene lists. **(I, b)** Enrichment analysis of the hub gene network. **(J, a)** Hub genes of PPI network (ranking third) and MCODE2 components identified in the gene lists. **(J, b)** enrichment analysis of the hub gene network.

For HEIH, a total of 7,066 HEIH DEGs were screened with threshold values of |log2-fold-change (FC)| >1.0 and *p*-adj (adjusted *P*-value) <0.05. A total of 2,567 lncRNAs, 210 miRNAs, 634 miscRNAs, 1,962 pseudogenes, and 568 protein-coding RNAs and other RNAs, including 490 downregulated genes and 78 upregulated genes, were identified and submitted to the following analysis (**Figure 4A**). GO and KEGG pathway enrichment analyses were conducted to identify the biological functions of the DEGs, and the BP, CC, MF, and KEGG terms were primarily involved in nucleosome assembly, nucleosome organization, nucleosome, DNA packaging complex, chromatin DNA binding, nucleosome binding, SLE, and alcoholism (**Figure 4B**). Then, to further dissect the function of the hub gene networks, the CytoHubba and mCODE plugins in Cytoscape were used to identify and extract the key subnetworks. The top three subnetworks are shown in **Figures 4Ca, Da, Ea**. Furthermore, function and pathway enrichment analysis of related genes in the three hub networks was performed using the DAVID database, followed by visualization in the form of separate bubble plots (**Figures 4Cb, Db, Eb**).

Next, a total of 4,457 YBX3 DEGs were acquired with the same screening settings as HEIH, including 1,417 lncRNAs, 124 miRNAs, 434 miscRNAs, 1,389 pseudogenes, 439 protein-coding RNAs, and other RNAs. Of a total of 439 genes, 186 upregulated genes and 253 downregulated genes were identified (**Figure 4F**). Similarly, the 439 genes were subjected to both GO and KEGG enrichment analyses. The results showed that the genes were mainly enriched in nucleosome assembly and chromatin assembly in the BP category, nucleosome and DNA packaging complex in the CC category, nucleosome binding and chromatin DNA binding in the MF category, and SLE and alcoholism in the KEGG analysis (**Figure 4G**). Then, the hub gene networks were acquired following the same methods as those used for HEIH, and interestingly, the results revealed highly similar top-ranked subnetworks for YBX3 and HEIH (**Figure 4Ha**). The second- and third-ranked hub gene subnetworks are presented in **Figures 4Ia, Ja**. Further GO and KEGG enrichment analysis was performed separately for hub genes in each hub gene subnetwork to investigate the roles of these genes in COAD; the results are visualized in three bubble plots (**Figures 4Hb, Ib, Jb**).

### 3.9 Localization, single-cell variation, and expression profile patterns of YBX3

**Figure 5:**
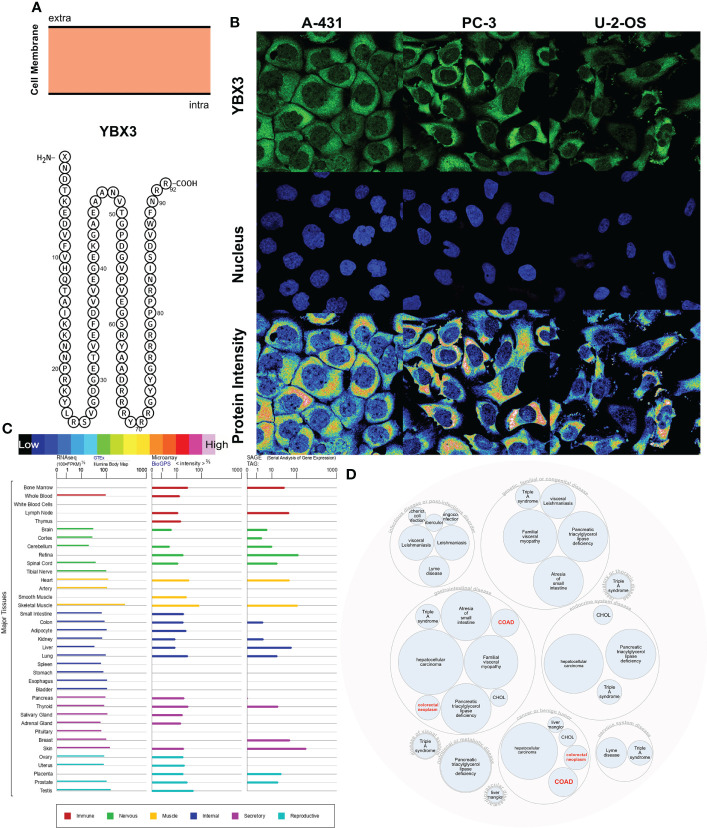
Localization, single-cell variation, and expression profile patterns of YBX3. **(A)** YBX3 protein topology. **(B)** Immunofluorescence staining of the subcellular distribution of YBX3 within the cytoplasm and nucleus of A-431, PC-3, and U-2 cell lines as adopted from the HPA database. **(C)** Bar plot of YBX3 mRNA expressions in diverse normal human tissues from the GTEx database. **(D)** Construction of YBX3-associated disease network.

The YBX3 protein topology analysis suggested intracellular membrane localization, as shown in **Figure 5A**. To further characterize the subcellular localization of YBX3, an indirect immunofluorescence assay in the A431, PC3, and U-2 OS cell lines was performed to evaluate the distribution of YBX3. YBX3 protein was expressed both in the cytoplasm and nucleus of the three cell lines, and the protein intensity was higher in the cytoplasm (**Figure 5B**). Moreover, we found that the YBX3 mRNA expression profile was moderate or higher in diverse normal human tissues, such as muscle system and secretory system tissues (**Figure 5C**). In **Figure 5D**, gene and disease interaction analysis indicated that YBX3 is associated with multiple diseases, especially cancers (hepatocellular carcinoma and colon cancer) and congenital diseases (triple A syndrome and familial visceral myopathy).

### 3.10 YBX3 expression is associated with various immune cell infiltration, T-cell exclusion, ICB subcohorts, and its opposite effect on HEIH in scRNA-seq chips

**Figure 6:**
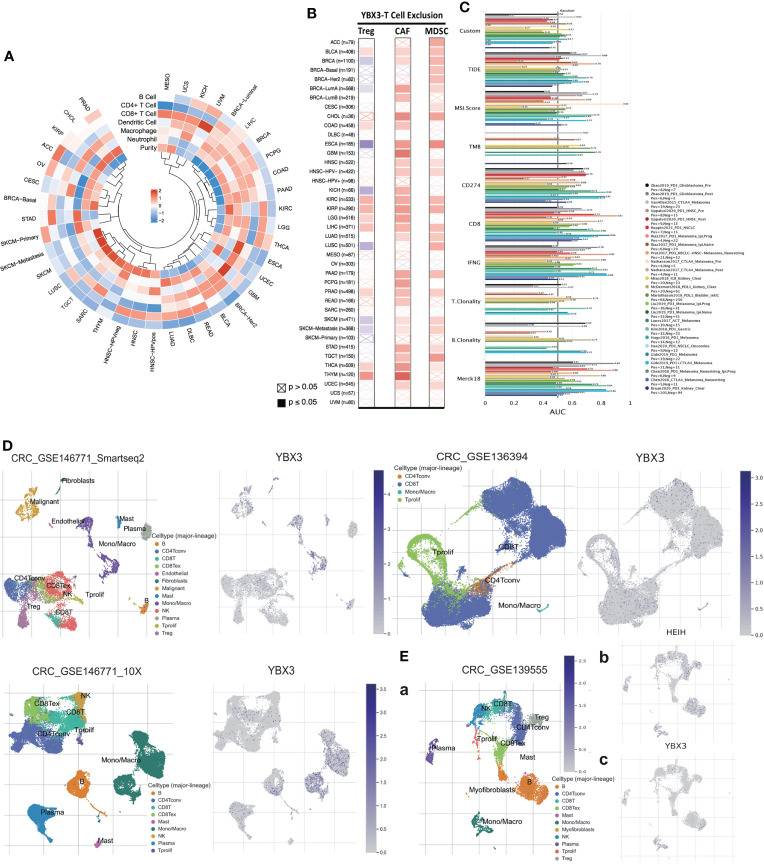
Impact of YBX3 expression on immune/immunosuppressive cells across The Cancer Genome Atlas (TCGA) cancers and on the biomarker value of ICB sub-cohorts and YBX3 expression profile in different immune cells. **(A)** Correlations between YBX3 expression and infiltration of six immune cell types across all cancer types in TCGA cohorts. **(B)** Correlations of YBX3 expression with three immunosuppressive cell types in various TCGA cancer types. **(C)** Biomarker relevance of MXD3 compared to standardized cancer immune evasion biomarkers in immune checkpoint blockade sub-cohorts. **(D, E)** Presenting YBX3 and HEIH expression profile in different immune cells using scRNA-seq data from GEO database.

Across 33 TCGA cancer types and six cancer subtypes, only LIHC showed a significantly positive correlation between YBX3 expression and the infiltration of all six immune cell types (neutrophils, macrophages, dendritic cells, CD8+ T cells, CD4+ T cells, and B cells). Interestingly, in COAD, YBX3 expression was significantly correlated with the infiltration of five immune cell types. In BRCA, PCPG, PAAD, LGG, THCA, and OV, the correlations between YBX3 expression and immune cell infiltration were significantly positive for four immune cell types. For other cancer types, YBX3 expression was significantly positively correlated with the infiltration of at least one type of immune cell (**Figure 6A** **)**.

The correlation between YBX3 expression and the infiltration of three immunosuppressive cell types, including Tregs and CAFs, which have been reported to promote T-cell exclusion among various cancer types, was also further evaluated. We found that YBX3 expression was positively correlated with the tumor infiltration of Tregs in BLCA, COAD, KIRC, KIRP, LGG, LIHC, PRAD, THCA, and THYM; tumor infiltration of CAFs in nearly all cancer types except ACC, BRCA (basal and Her2 molecular subtypes), CESC, DLBC, (HPV+)HNSC, LUSC, MESO, OV, SARC, UCS, and UVM; and tumor infiltration of MDSCs in ACC, BLCA, BRCA (except LumB molecular subtypes), CESC, CHOL, ESCA, HNSC (without HPV+), KIRC, KIRP, LIHC, LUAD, LUSC, PAAD, SKCM, STAD, TGCT, THCA, and UCEC (**Figure 6B**).

The relevance of YBX3 as a biomarker was also assessed by comparing it with standardized biomarkers in terms of their ability to predict clinical outcomes in ICB subcohorts (**Figure 6C**). We observed that YBX3 alone had an AUC of ≥0.5 in nine of the 24 ICB subcohorts. MXD3 presented a higher predictive value than TMB and B clonality, which showed AUC values of >0.5 in eight and seven ICB subcohorts, respectively. Nevertheless, MXD3 was comparable to T clonality (AUC >0.5 in nine ICB subcohorts) but lower than TIDE, MSI score, CD274, CD8, IFNG, and Merck18.

After visualizing the scRNA-seq data, we analyzed data from colon cancer chips, such as 146771 and 136394, and found that YBX3 was highly expressed in various cell types, especially monocytic series, malignant cells, and CD8 T cells (**Figure 6D**). In another chip, 139555 (**Figure 6Ea**), surprisingly, we found that YBX3 expression was very low in cell lineages with a relatively high HEIH expression (**Figure 6Eb**), for example, CD4 Tconv, plasma cells, NK cells, and B cells, presenting a distinctly opposite trend (**Figure 6Ec**).

### 3.11 YBX3 expression correlates with therapeutic responses among various cancer types

We observed that among breast and ovarian cancer cohorts, a higher YBX3 expression was significantly associated with resistance to chemotherapies (both *P <*0.01). However, glioma and colon cancer patients with a higher YBX3 expression tended to benefit more from chemotherapy than those with a lower YBX3 expression, with *P*-values of 0.065 and 0.011 for glioma and colon cancer, respectively (**Supplementary Figure S1–5A**).

Furthermore, we observed that lower expression levels of YBX3 were associated with improved clinical outcomes for ICB therapy (PDL1 or PD1) in bladder cancer and glioblastomas and consequently longer survival times (**Supplementary Figure S1–5B**, upper row). Conversely, a higher YBX3 expression was associated with more benefits of CTLA4 ICB in melanoma patients and thus a prolonged survival period. Moreover, in melanoma cohorts, a higher expression of YBX3 was associated with a higher level of CTLs, indicating an interplay with CTL exclusion (**Supplementary Figure S1–5B**, lower row).

### 3.12 Experimental validation of the expression and relationship of the HEIH/YBX3 regulatory system in colon cancer

#### 3.12.1 YBX3 protein, mediating a variety of functions in colon cancer, was eluted in HEIH RNA pull-down assay, confirmed by MS, and upregulated after HEIH knockdown

**Figure 7:**
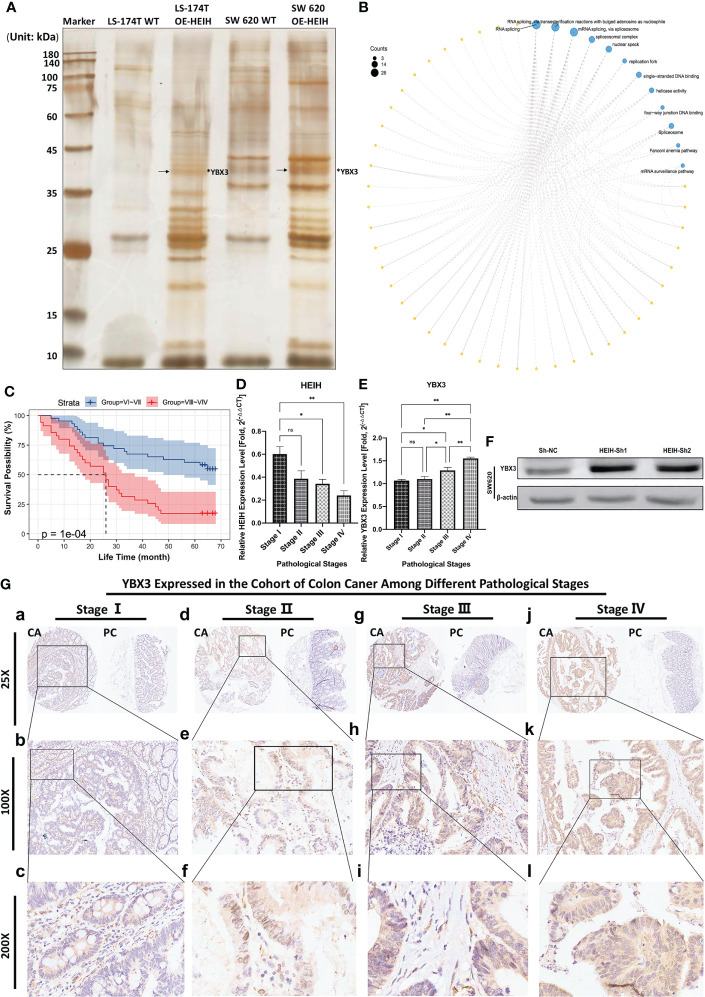
Experimental verification of the combination and role of HEIH/YBX3 in colon cancer. **(A)** lncRNA HEIH pull-down. **(B)** Enrichment analysis of the pulled-down proteins. **(C)** Correlation between YBX3 expression in diverse clinical stages and prognosis in the colon cancer cohort. **(D)** HEIH expression level in various cancer stages quantified by qPCR. **(E)** YBX3 expression level in various cancer stages quantified by qPCR. **(F)** In SW620 cell line, lncRNA-HEIH knockdown enhanced the YBX3 expression confirmed by western blot. **(G)** Immunohistochemical staining of tissue microarray showing the YBX3 expression in different clinical pathological stages in magnifications of ×25, ×100, and ×200, respectively. *P<0.05; **P<0.01; ns, non-significant.

As **Figure 7A** shows, in this RNA pull-down assay, the product obtained from positive-sense single-stranded HEIH RNA was used as the bait in the experimental group, while the biotin-NC probe, the negative control probe labeled with biotin, was used as a control. Significant differences between the HEIH RNA experimental group and the biotin-labeled control groups were found. Given that the samples did not show high protein abundance, liquid chromatography–mass spectrometry/mass spectrometry was directly performed to analyze the eluates, and YBX3 protein was identified (marked with arrows). After comparing the spectrometry map and peptide alignment, 98 additional proteins were identified by peptide profile matching; all the details are listed in **Supplementary File 2**. To explore the functions of the identified proteins, GO and KEGG enrichment analyses were performed to identify their roles in the carcinogenesis of colon cancer. The results revealed that “RNA splicing”, “RNA splicing *via* transesterification reactions with bulged adenosine as nucleophile”, and “mRNA splicing *via* spliceosome” were the top three enriched BP terms; “spliceosome complex”, “nuclear speck”, and “replication fork” were the enriched CC terms; “single-stranded DNA binding”, “helicase activity”, and “four-way junction DNA binding” were the enriched MF terms; and finally, the enriched KEGG pathways were “spliceosome”, “Fanconi anemia pathway”, and “mRNA surveillance pathway” (**Figure 7B**). Furthermore, WB demonstrated that lncRNA-HEIH knockdown enhanced YBX3 expression in the SW620 cell line (**Figure 7F**).3.12.2 YBX3 was highly expressed in the colon cancer TMA and significantly affected patient prognosis, with increased expression in cDNA samples with increasing tumor stage

In our own TMA cohort, immunohistochemistry (IHC) staining of the specimens showed that the expression level of YBX3 increased with increasing pathological stage, which was consistent with the staining scores. The higher/lower stage groups were defined according to pathological stages: V I and V II composed the lower stage group, while V III and V IV composed the higher stage group. Then, a survival curve was plotted based on the collected survival data of the cohort, showing that patients with lower stages (namely, low YBX3 expression level) had better outcomes than those patients with higher stages, with *P*-value = 10e-4 (**Figure 7C**). We performed a qPCR analysis of cDNA samples from 12 patients from our hospital with distinct stages of colon cancer, and we found that, as the tumor stage progressed, HEIH expression decreased (**Figure 7D**), while YBX3 expression rose (**Figure 7E**). IHC staining of the TMA showed that the expression of YBX3 was significantly greater in colon cancer tissues than in paracancerous tissues, and the trend was more evident in the cytoplasm of glandular cells and colonic epithelial cells. Moreover, the protein expression level of YBX3 gradually increased with cancer stage and pathological grade (**Figure 7F**).

## 4. Discussion

The upregulation of lncRNA-HEIH was first observed in hepatocellular carcinoma (15); subsequently, its role in gastric adenocarcinoma, melanoma, non-small-cell lung cancer, ovarian cancer, nasopharyngeal cancer, and esophageal cancer was successively discovered, and lncRNA-HEIH showed an upregulated pattern in all of these tumors (16–20). Similarly, YBX genes, which are members of a transcription factor family that is expressed predominantly in endothelial cells, are essential not only in the regulation of cell proliferation and differentiation but also in regulating the expression of TJ proteins, especially in promoting HCC progression and KIRC proliferation and regulating the level of TJ proteins in melanoma (21–23). However, research on YBX3, an emerging member of the YBX family, in cancer development is limited. To date, studies of YBX3 have been reported only for breast, liver, and kidney cancers (24–26). Hence, to our knowledge, no existing studies have fully described the significance of HEIH and YBX3 across cancers. Unexpectedly, our research found that the lncRNA HEIH, which has been reported several times to be highly expressed in multiple tumors (27), showed the opposite trend in colon cancer (serving as a tumor suppressor gene). Previously, we used the Ensemble and RBPsuite databases to predict the interaction of the lncRNA HEIH and the YBX3 protein. We divided this lncRNA, which is 1,141 bp in length, into 11 segments of 101 bp each and found that each of these segments is predicted to bind to the YBX3 protein with a likelihood of more than 50%, reaching as high as 99.37% (**Supplementary File 3**), and most of the combined regions were located in the 3’-terminal. This surprising result implies that it is necessary to deeply explore the relationship between these factors as well as the tumor regulatory system that they represent across cancers.

When we evaluated the diagnostic values of HEIH/YBX3 across cancers among TCGA samples, we found that HEIH played a significant diagnostic role in eight of the 10 cancer types where YBX3 played a significant diagnostic role (AUC > 0.8). Furthermore, the AUC values of the two factors were quite similar, which showed the reliability and similarity of HEIH and YBX3 in cancer diagnosis. Moreover, by studying the significance of HEIH and YBX3 on the prognosis of digestive system tumors, we found that they had completely opposite prognostic relationships with the same tumor types, suggesting that there is a suppressive effect between HEIH and YBX3. Next, after using the RNA expression profiles of 521 samples in the TCGA-COAD cohort to find the DEGs associated with HEIH and YBX3 and constructing their respective hub gene networks, we were surprised to find that, of the three hub gene networks with the highest scores for each gene, two were almost the same. To further explore the functional roles of these two genes in the regulation of colon cancer, we performed enrichment analysis of the whole hub gene networks and found that the main GO and KEGG enrichment results were very similar and involved nucleosome assembly, DNA packaging complex, and nucleosome binding, suggesting that these genes might play an important role in DNA replication or epigenetics, especially in chromatin remodeling. Furthermore, another direct evidence was the immune repertoire drawn by scRNA-seq results from GSE chips. An obviously opposite expression trend was also observed between HEIH and YBX3 in the same types of immune cells, suggesting that HEIH exerts negative feedback regulation on YBX3 and that the close relationship between the two can constitute a HEIH/YBX3 regulatory system. In addition, in order to clarify the intrinsic logic line of the manuscript, we drew a flowchart to present the thread more intuitively (**Supplementary Figure S1–6**).

Our results also indicated that YBX3 is a well-conserved protein, and the protein topology predicted membrane localization without any variants. Immunofluorescence staining also showed its subcellular location, and the distribution of the YBX3 protein in both the nucleus and the cytoplasm indicated its extensive molecular functions. As previously mentioned, the relationships of YBX3 expression levels with tumor staging, metastasis, tumor microenvironment, immune evasion, and chemotherapeutic drug sensitivity across 39 TCGA cancer types and subtypes were further investigated. These findings suggest that YBX3 plays a role in driving tumor progression, lymphatic invasion, and metastasis in distinct cancers, especially in digestive tract cancers, such as ESAD, STAD, and COAD. Moreover, for patients suffering from colon cancer with a higher degree of malignancy, a higher expression of YBX3 predicts worse prognosis, which demonstrates that it is associated with high-risk clinical features. However, the results of lncRNA-HEIH were diametrically opposite, which indicated the opposite effect of the two genes in terms of clinical significance.

Previous studies have reported that some oncogenic proteins, such as KPNA2, can regulate the tumor infiltration of immune cells (28). Other recent studies have proven that the TGF-β1 secreted by CAFs significantly enhanced the level of Ln-γ2, which is encoded by LAMC2 *via* c-Jun N-terminal kinase signaling in tumor cells to prevent access of T cells to the tumor nest, that Treg cells exerted negative regulatory effects on immune responses caused by the tumor through cell-to-cell contact with various immune cell subsets and by secreting inhibitory cytokines, and that MDSCs can upregulate the activity of Arg-1, thus leaving immune cells in the unresponsive and tolerant state, which mediates the dysfunction of T cells (29–31). However, during the past 10 years, the issue regarding the infiltration of CAFs and the immunotherapeutic effect driven by YBX3 has been seldom studied due to the novelty of the protein. Recently, research centering on immune cell infiltration induced by YBX3, especially in TAMs, T-cell exclusion, and TME, has been gradually emerging, such as those of Dora *et al.* and Carreras *et al.* (32, 33), with the help of multilayer perceptron artificial neural network and CIBERSORT abs.mode. Our research, however, found that, across 39 TCGA databases, YBX3 is associated with tumor infiltration of all kinds of immune cells only in LIHC, indicating that YBX3 might enhance tumor immune evasion and progression of LIHC through dysfunctional T-cell phenotypes. Furthermore, in tumors such as BLCA, COAD, KIRC, KIRP, LGG, LIHC, PRAD, and THCA, the expression of YBX3 can significantly promote T-cell exclusion, which results in resistance to checkpoint blockade. Moreover, in this study it was observed that, in some cancer cohorts, such as bladder cancer, a lower YBX3 expression was associated with a greater infiltration of CTLs. This leads to the clinical phenomenon that patients who have a low expression level of YBX3 tend to be more sensitive to immune checkpoint blockade therapy, suggesting that YBX3 expression endows cancer cells with a more versatile regulatory response that enables them to evade immune checkpoint inhibitors.

## 5. Conclusion

The characterization of YBX3 and its regulatory lncRNA HEIH in terms of diagnostic and prognostic significance, the immuno-oncologic context of the TME and therapeutic responses across cancers, and especially further exploring them in colon cancer both experimentally and bioinformatically add another dimension to our understanding of their critical roles in tumor progression and suppression and offer an experimental and integrative basis for deeper verification of their molecular biology and even their future clinical application in cancer therapies. Our findings suggest that the oncogenic system of HEIH/YBX3 could together serve as a biomarker for cancer detection, prognosis, therapy design, and follow-up.

## Data availability statement

The original contributions presented in the study are included in the article/**Supplementary Material**. Further inquiries can be directed to the corresponding authors.

## Ethics statement

This study was reviewed and approved by Clinical Ethics Committee of the Second Affiliated Hospital of Army Medical University of the PLA (NO: 2022-036-01). The patients/participants provided their written informed consent to participate in this study.

## Author contributions

YS: conceptualization, methodology, software, formal analysis, writing—original draft, experiment, and visualization. ZL: conceptualization, methodology, visualization, supervision, and experiment. WW: software, formal analysis, and writing—original draft. XZ: software, validation, and investigation. WL: conceptualization, methodology, visualization, and supervision. GD: software, validation, and investigation. JY: software, formal analysis, writing—original draft, visualization, software, and investigation. WX: software, formal analysis, writing—original draft, visualization, software, and investigation. HY: conceptualization, methodology, writing—review supervision, and funding acquisition. All authors contributed to the article and approved the submitted version.

## Funding

This work was supported by the National Natural Science Foundation of China (grant numbers 81873551 and 81270576).

## Conflict of interest

The authors declare that the research was conducted in the absence of any commercial or financial relationships that could be construed as a potential conflict of interest.

## Publisher’s note

All claims expressed in this article are solely those of the authors and do not necessarily represent those of their affiliated organizations, or those of the publisher, the editors and the reviewers. Any product that may be evaluated in this article, or claim that may be made by its manufacturer, is not guaranteed or endorsed by the publisher.
