# Supplementary material for: Identification and verification of YBX3 and its regulatory gene HEIH as an oncogenic system: A multidimensional analysis in colon cancer
Source: Front Immunol. 2022 Aug 18;13:957865. doi: 10.3389/fimmu.2022.957865 (PMC9433931; doi:10.3389/fimmu.2022.957865)
Supplement: Supplementary file 2 [file Table_1.docx]

| Characteristic | Low expression of YBX3 | High expression of YBX3 | p |
| --- | --- | --- | --- |
| n | 239 | 239 |  |
| T stage, n (%) |  |  | 0.072 |
| T1 | 8 (1.7%) | 3 (0.6%) |  |
| T2 | 47 (9.9%) | 36 (7.5%) |  |
| T3 | 161 (33.8%) | 162 (34%) |  |
| T4 | 23 (4.8%) | 37 (7.8%) |  |
| N stage, n (%) |  |  | 0.157 |
| N0 | 149 (31.2%) | 135 (28.2%) |  |
| N1 | 55 (11.5%) | 53 (11.1%) |  |
| N2 | 35 (7.3%) | 51 (10.7%) |  |
| M stage, n (%) |  |  | 0.027 |
| M0 | 187 (45.1%) | 162 (39%) |  |
| M1 | 25 (6%) | 41 (9.9%) |  |
| Age, meidan (IQR) | 68 (58, 75.5) | 69 (58.5, 78) | 0.429 |

**The baseline information of YBX3 expression in colon cancer cohorts**
