# Supplementary material for: Identification and verification of YBX3 and its regulatory gene HEIH as an oncogenic system: A multidimensional analysis in colon cancer
Source: Front Immunol. 2022 Aug 18;13:957865. doi: 10.3389/fimmu.2022.957865 (PMC9433931; doi:10.3389/fimmu.2022.957865)
Supplement: Supplementary file 3 [file Table_2.docx]

| Characteristic | Low expression of HEIH | High expression of HEIH | p |
| --- | --- | --- | --- |
| n | 239 | 239 |  |
| T stage, n (%) |  |  | 0.399 |
| T1 | 4 (0.8%) | 7 (1.5%) |  |
| T2 | 37 (7.8%) | 46 (9.6%) |  |
| T3 | 170 (35.6%) | 153 (32.1%) |  |
| T4 | 28 (5.9%) | 32 (6.7%) |  |
| N stage, n (%) |  |  | 0.108 |
| N0 | 152 (31.8%) | 132 (27.6%) |  |
| N1 | 45 (9.4%) | 63 (13.2%) |  |
| N2 | 42 (8.8%) | 44 (9.2%) |  |
| M stage, n (%) |  |  | 0.777 |
| M0 | 170 (41%) | 179 (43.1%) |  |
| M1 | 34 (8.2%) | 32 (7.7%) |  |
| Age, meidan (IQR) | 69 (58.5, 76) | 68 (58, 78) | 0.839 |

**The baseline information of lncRNA-HEIH expression in colon cancer cohorts**
