# Supplementary material for: Identification and verification of YBX3 and its regulatory gene HEIH as an oncogenic system: A multidimensional analysis in colon cancer
Source: Front Immunol. 2022 Aug 18;13:957865. doi: 10.3389/fimmu.2022.957865 (PMC9433931; doi:10.3389/fimmu.2022.957865)
Supplement: Supplementary file 4 [file Table_3.docx]

| Characteristics  Univariate/Multivariate cox regression analysis of overall survival in colon cancer cohorts | Total(N) | Univariate analysis | |  | Multivariate analysis | |
| --- | --- | --- | --- | --- | --- | --- |
|  |  | Hazard ratio (95% CI) | P value |  | Hazard ratio (95% CI) | P value |
| T stage | 476 |  |  |  |  |  |
| T1 | 11 | Reference |  |  |  |  |
| T2 | 83 | 0.417 (0.080-2.159) | 0.297 |  | 160337402526329225961537536.000 (0.000-Inf) | 1.000 |
| T3 | 322 | 1.291 (0.316-5.268) | 0.722 |  | 2448398631972267008.000 (0.000-Inf) | 1.000 |
| T4 | 60 | 3.508 (0.818-15.037) | 0.091 |  | 0.011 (0.000-Inf) | 1.000 |
| N stage | 477 |  |  |  |  |  |
| N0 | 283 | Reference |  |  |  |  |
| N1 | 108 | 1.681 (1.019-2.771) | **0.042** |  | 0.000 (0.000-Inf) | 1.000 |
| N2 | 86 | 4.051 (2.593-6.329) | **<0.001** |  | 0.000 (0.000-Inf) | 1.000 |
| M stage | 414 |  |  |  |  |  |
| M0 | 348 | Reference |  |  |  |  |
| M1 | 66 | 4.193 (2.683-6.554) | **<0.001** |  | 143417351816202633216.000 (0.000-Inf) | 1.000 |
| ERBB2 | 477 |  |  |  |  |  |
| Low | 239 | Reference |  |  |  |  |
| High | 238 | 0.990 (0.672-1.459) | 0.959 |  |  |  |
| TP53 | 477 | 0.908 (0.765-1.077) | 0.268 |  |  |  |
| Pathologic stage | 466 |  |  |  |  |  |
| Stage I | 81 | Reference |  |  |  |  |
| Stage II | 186 | 2.035 (0.785-5.273) | 0.143 |  | 39610941.324 (0.000-Inf) | 1.000 |
| Stage III | 133 | 3.683 (1.436-9.448) | **0.007** |  | 639.324 (0.000-Inf) | 1.000 |
| Stage IV | 66 | 9.294 (3.608-23.936) | **<0.001** |  | 1.000 (0.000-Inf) | 1.000 |
| Primary therapy outcome | 250 |  |  |  |  |  |
| PD&SD | 29 | Reference |  |  |  |  |
| PR | 13 | 0.272 (0.062-1.189) | 0.084 |  | 1.000 (1.000-1.000) |  |
| CR | 208 | 0.088 (0.045-0.172) | **<0.001** |  | 0.000 (0.000-Inf) | 1.000 |
| Gender | 477 |  |  |  |  |  |
| Female | 226 | Reference |  |  |  |  |
| Male | 251 | 1.101 (0.746-1.625) | 0.627 |  |  |  |
| Race | 306 |  |  |  |  |  |
| Asian | 11 | Reference |  |  |  |  |
| Black or African American | 63 | 0.927 (0.208-4.133) | 0.921 |  |  |  |
| White | 232 | 0.810 (0.196-3.346) | 0.771 |  |  |  |
| Age | 477 |  |  |  |  |  |
| <=65 | 194 | Reference |  |  |  |  |
| >65 | 283 | 1.610 (1.052-2.463) | **0.028** |  | 0.000 (0.000-Inf) | 1.000 |
| Weight | 273 |  |  |  |  |  |
| <=90 | 189 | Reference |  |  |  |  |
| >90 | 84 | 0.601 (0.292-1.238) | 0.167 |  |  |  |
| Height | 256 |  |  |  |  |  |
| <170 | 127 | Reference |  |  |  |  |
| >=170 | 129 | 0.786 (0.445-1.389) | 0.407 |  |  |  |
| BMI | 256 |  |  |  |  |  |
| <25 | 87 | Reference |  |  |  |  |
| >=25 | 169 | 0.549 (0.311-0.969) | **0.038** |  | 11.363 (0.000-Inf) | 1.000 |
| Residual tumor | 373 |  |  |  |  |  |
| R0 | 345 | Reference |  |  |  |  |
| R1&R2 | 28 | 4.364 (2.401-7.930) | **<0.001** |  | 2558196506007739904.000 (0.000-Inf) | 1.000 |
| CEA level | 302 |  |  |  |  |  |
| <=5 | 195 | Reference |  |  |  |  |
| >5 | 107 | 3.128 (1.788-5.471) | **<0.001** |  | 0.001 (0.000-Inf) | 1.000 |
| Perineural invasion | 181 |  |  |  |  |  |
| NO | 135 | Reference |  |  |  |  |
| YES | 46 | 1.940 (0.982-3.832) | 0.056 |  | 6.816 (0.000-Inf) | 1.000 |
| Lymphatic invasion | 433 |  |  |  |  |  |
| NO | 265 | Reference |  |  |  |  |
| YES | 168 | 2.450 (1.614-3.720) | **<0.001** |  | 870758.099 (0.000-Inf) | 1.000 |
| History of colon polyps | 407 |  |  |  |  |  |
| NO | 262 | Reference |  |  |  |  |
| YES | 145 | 0.741 (0.442-1.242) | 0.255 |  |  |  |
