# Supplementary material for: Identification and verification of YBX3 and its regulatory gene HEIH as an oncogenic system: A multidimensional analysis in colon cancer
Source: Front Immunol. 2022 Aug 18;13:957865. doi: 10.3389/fimmu.2022.957865 (PMC9433931; doi:10.3389/fimmu.2022.957865)
Supplement: Supplementary file 5 [file Table_4.docx]

| Characteristics | Total(N) | Univariate analysis  Univariate/Multivariate cox regression analysis of disease specific survival in colon cancer cohorts | |  | Multivariate analysis | |
| --- | --- | --- | --- | --- | --- | --- |
|  |  | Hazard ratio (95% CI) | P value |  | Hazard ratio (95% CI) | P value |
| T stage | 460 |  |  |  |  |  |
| T1 | 11 | Reference |  |  |  |  |
| T2 | 82 | 0.148 (0.009-2.370) | 0.177 |  | 0.591 (0.000-Inf) | 1.000 |
| T3 | 307 | 1.543 (0.212-11.220) | 0.669 |  | 0.000 (0.000-Inf) | 1.000 |
| T4 | 60 | 5.190 (0.690-39.024) | 0.110 |  | 0.000 (0.000-Inf) | 1.000 |
| N stage | 461 |  |  |  |  |  |
| N0 | 275 | Reference |  |  |  |  |
| N1 | 105 | 2.601 (1.353-5.000) | **0.004** |  | 5280350.772 (0.000-Inf) | 1.000 |
| N2 | 81 | 6.357 (3.512-11.504) | **<0.001** |  | 1726345215570763518574592.000 (1726345215570763518574592.000-1726345215570763518574592.000) | **<0.001** |
| M stage | 399 |  |  |  |  |  |
| M0 | 334 | Reference |  |  |  |  |
| M1 | 65 | 7.833 (4.597-13.346) | **<0.001** |  | 0.000 (0.000-Inf) | 1.000 |
| ERBB2 | 461 |  |  |  |  |  |
| Low | 228 | Reference |  |  |  |  |
| High | 233 | 0.952 (0.582-1.555) | 0.843 |  |  |  |
| TP53 | 461 | 0.984 (0.787-1.231) | 0.890 |  |  |  |
| Pathologic stage | 451 |  |  |  |  |  |
| Stage I | 81 | Reference |  |  |  |  |
| Stage II | 178 | 2.058 (0.450-9.407) | 0.352 |  | 111591484.850 (0.000-Inf) | 1.000 |
| Stage III | 127 | 5.775 (1.343-24.830) | **0.018** |  | 43.193 (0.000-Inf) | 1.000 |
| Stage IV | 65 | 22.847 (5.455-95.699) | **<0.001** |  | 1.000 (0.000-Inf) | 1.000 |
| Primary therapy outcome | 249 |  |  |  |  |  |
| PD&SD | 28 | Reference |  |  |  |  |
| PR | 13 | 0.260 (0.059-1.146) | 0.075 |  | 1.000 (1.000-1.000) |  |
| CR | 208 | 0.034 (0.013-0.087) | **<0.001** |  | 0.000 (0.000-Inf) | 1.000 |
| Gender | 461 |  |  |  |  |  |
| Female | 220 | Reference |  |  |  |  |
| Male | 241 | 1.142 (0.697-1.871) | 0.599 |  |  |  |
| Race | 290 |  |  |  |  |  |
| Asian | 11 | Reference |  |  |  |  |
| Black or African American | 63 | 1.439 (0.184-11.255) | 0.729 |  |  |  |
| White | 216 | 0.702 (0.094-5.263) | 0.731 |  |  |  |
| Age | 461 |  |  |  |  |  |
| <=65 | 191 | Reference |  |  |  |  |
| >65 | 270 | 1.165 (0.702-1.933) | 0.555 |  |  |  |
| Weight | 258 |  |  |  |  |  |
| <=90 | 174 | Reference |  |  |  |  |
| >90 | 84 | 0.907 (0.380-2.162) | 0.825 |  |  |  |
| Height | 241 |  |  |  |  |  |
| <170 | 122 | Reference |  |  |  |  |
| >=170 | 119 | 0.658 (0.284-1.525) | 0.329 |  |  |  |
| BMI | 241 |  |  |  |  |  |
| <25 | 79 | Reference |  |  |  |  |
| >=25 | 162 | 0.979 (0.415-2.310) | 0.961 |  |  |  |
| Residual tumor | 373 |  |  |  |  |  |
| R0 | 345 | Reference |  |  |  |  |
| R1&R2 | 28 | 6.107 (3.225-11.563) | **<0.001** |  | 32.533 (32.533-32.533) | **<0.001** |
| CEA level | 301 |  |  |  |  |  |
| <=5 | 194 | Reference |  |  |  |  |
| >5 | 107 | 3.018 (1.543-5.901) | **0.001** |  | 127.725 (0.000-Inf) | 1.000 |
| Perineural invasion | 180 |  |  |  |  |  |
| NO | 134 | Reference |  |  |  |  |
| YES | 46 | 2.977 (1.325-6.686) | **0.008** |  | 12389774.409 (0.000-Inf) | 1.000 |
| Lymphatic invasion | 422 |  |  |  |  |  |
| NO | 255 | Reference |  |  |  |  |
| YES | 167 | 4.133 (2.361-7.235) | **<0.001** |  | 0.800 (0.000-Inf) | 1.000 |
| History of colon polyps | 396 |  |  |  |  |  |
| NO | 253 | Reference |  |  |  |  |
| YES | 143 | 0.907 (0.497-1.657) | 0.752 |  |  |  |
