# Supplementary material for: Identification and verification of YBX3 and its regulatory gene HEIH as an oncogenic system: A multidimensional analysis in colon cancer
Source: Front Immunol. 2022 Aug 18;13:957865. doi: 10.3389/fimmu.2022.957865 (PMC9433931; doi:10.3389/fimmu.2022.957865)
Supplement: Supplementary file 1 [file Image_1.pdf]

**Fig. S1-1**

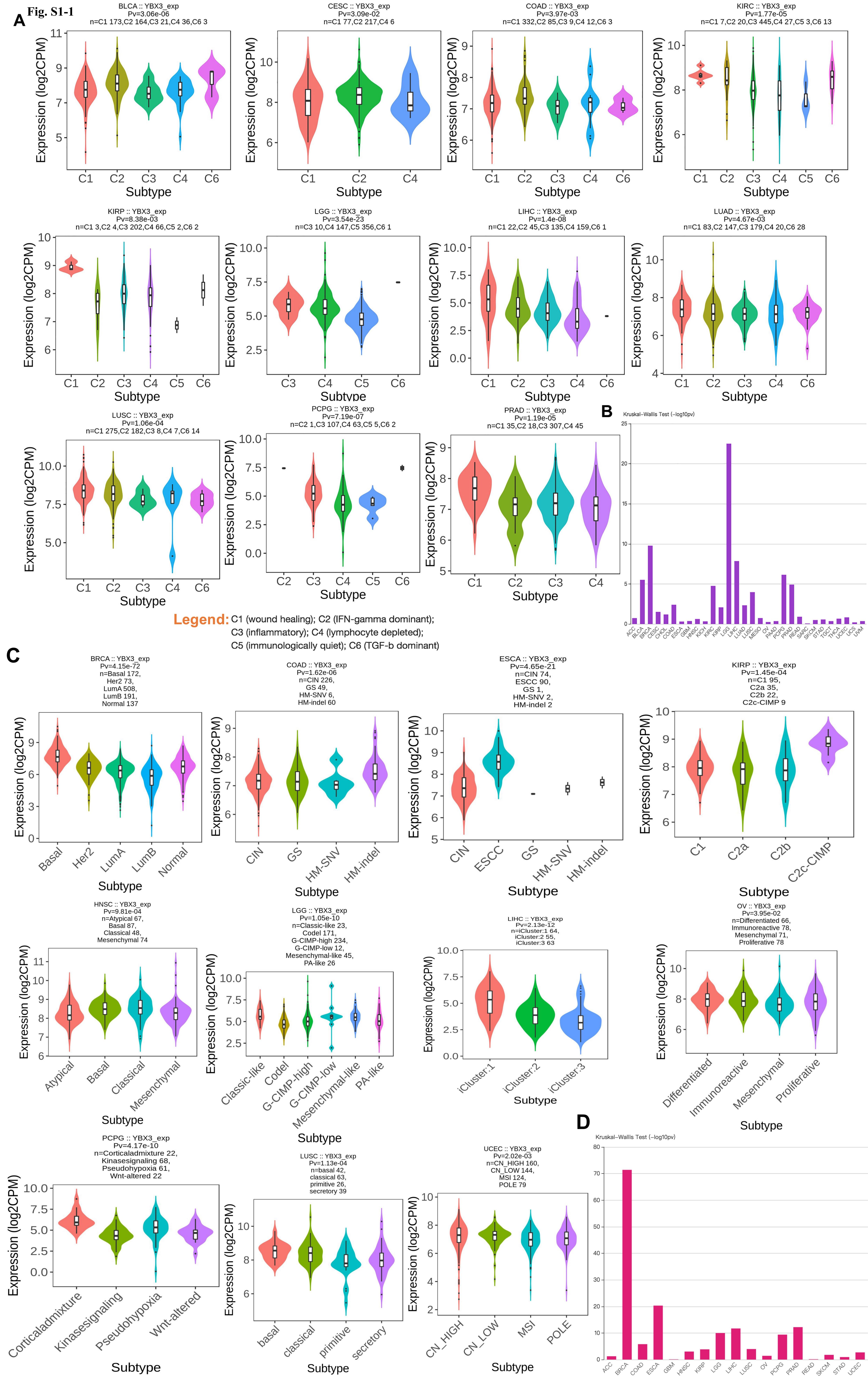

A
BLCA :: YBX3_exp
Pv=3.06e-06
n=C1 173,C2 164,C3 21,C4 36,C6 3
CESC :: YBX3_exp
Pv=3.09e-02
n=C1 77,C2 217,C4 6
COAD :: YBX3_exp
Pv=3.97e-03
n=C1 332,C2 85,C3 9,C4 12,C6 3
KIRC :: YBX3_exp
Pv=1.77e-05
n=C1 7,C2 20,C3 445,C4 27,C5 3,C6 13
KIRP :: YBX3_exp
Pv=8.38e-03
n=C1 3,C2 4,C3 202,C4 66,C5 2,C6 2
LGG :: YBX3_exp
Pv=3.54e-23
n=C3 10,C4 147,C5 356,C6 1
LIHC :: YBX3_exp
Pv=1.4e-08
n=C1 22,C2 45,C3 135,C4 159,C6 1
LUAD :: YBX3_exp
Pv=4.67e-03
n=C1 83,C2 147,C3 179,C4 20,C6 28
LUSC :: YBX3_exp
Pv=1.06e-04
n=C1 275,C2 182,C3 8,C4 7,C6 14
PCPG :: YBX3_exp
Pv=7.19e-07
n=C2 1,C3 107,C4 63,C5 5,C6 2
PRAD :: YBX3_exp
Pv=1.19e-05
n=C1 35,C2 18,C3 307,C4 45
Expression (log2CPM)
Subtype
Legend: C1 (wound healing); C2 (IFN-gamma dominant); C3 (inflammatory); C4 (lymphocyte depleted); C5 (immunologically quiet); C6 (TGF-b dominant)
B
Kruskal-Wallis Test (-log10pv)
C
BRCA :: YBX3_exp
Pv=4.15e-72
n=Basal 172, Her2 73, LumA 508, LumB 191, Normal 137
COAD :: YBX3_exp
Pv=1.62e-06
n=CIN 226, GS 49, HM-SNV 6, HM-indel 60
ESCA :: YBX3_exp
Pv=4.65e-21
n=CIN 74, ESCC 90, GS 1, HM-SNV 2, HM-indel 2
KIRP :: YBX3_exp
Pv=1.45e-04
n=C1 95, C2a 35, C2b 22, C2c-CIMP 9
HNSC :: YBX3_exp
Pv=9.81e-04
n=Atypical 67, Basal 87, Classical 48, Mesenchymal 74
LGG :: YBX3_exp
Pv=1.05e-10
n=Classic-like 23, Codel 171, G-CIMP-high 234, G-CIMP-low 12, Mesenchymal-like 45, PA-like 26
LIHC :: YBX3_exp
Pv=2.13e-12
n=iCluster:1 64, iCluster:2 55, iCluster:3 63
OV :: YBX3_exp
Pv=3.95e-02
n=Differentiated 66, Immunoreactive 78, Mesenchymal 71, Proliferative 78
PCPG :: YBX3_exp
Pv=4.17e-10
n=Corticaladmixture 22, Kinasesignaling 68, Pseudohypoxia 61, Wnt-altered 22
LUSC :: YBX3_exp
Pv=1.13e-04
n=basal 42, classical 63, primitive 26, secretory 39
UCEC :: YBX3_exp
Pv=2.02e-03
n=CN_HIGH 160, CN_LOW 144, MSI 124, POLE 79
D
Kruskal-Wallis Test (-log10pv)

Fig. S1-2

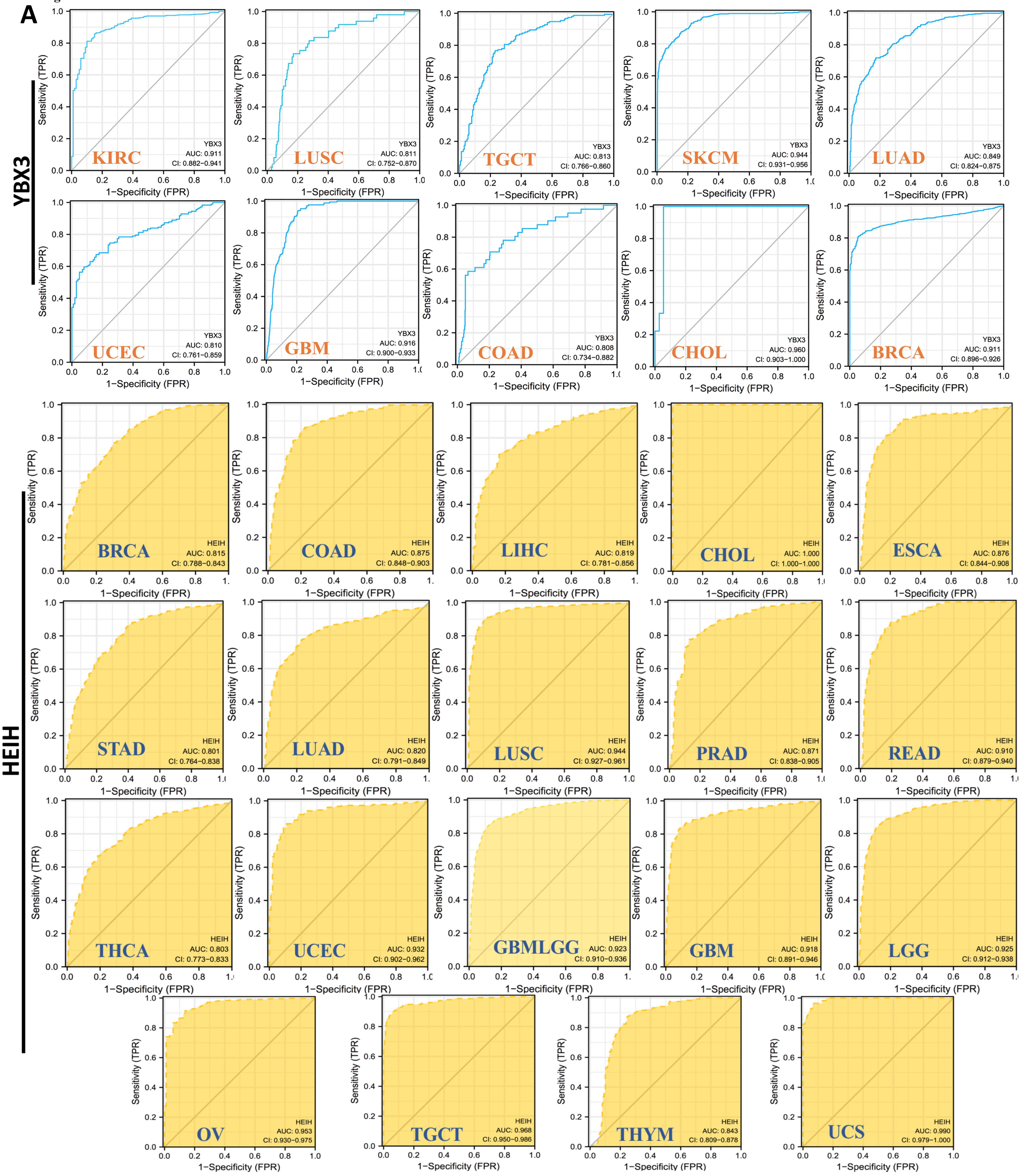
A
YBX3
HEIH
Sensitivity (TPR)
1−Specificity (FPR)
KIRC
YBX3
AUC: 0.911
CI: 0.882−0.941
LUSC
YBX3
AUC: 0.811
CI: 0.752−0.870
TGCT
YBX3
AUC: 0.813
CI: 0.766−0.860
SKCM
YBX3
AUC: 0.944
CI: 0.931−0.956
LUAD
YBX3
AUC: 0.849
CI: 0.824−0.875
UCEC
YBX3
AUC: 0.810
CI: 0.761−0.859
GBM
YBX3
AUC: 0.916
CI: 0.900−0.933
COAD
YBX3
AUC: 0.808
CI: 0.734−0.882
CHOL
YBX3
AUC: 0.960
CI: 0.903−1.000
BRCA
YBX3
AUC: 0.911
CI: 0.896−0.926
BRCA
HEIH
AUC: 0.815
CI: 0.788−0.843
COAD
HEIH
AUC: 0.875
CI: 0.848−0.903
LIHC
HEIH
AUC: 0.819
CI: 0.781−0.856
CHOL
HEIH
AUC: 1.000
CI: 1.000−1.000
ESCA
HEIH
AUC: 0.876
CI: 0.844−0.908
STAD
HEIH
AUC: 0.801
CI: 0.764−0.838
LUAD
HEIH
AUC: 0.820
CI: 0.791−0.849
LUSC
HEIH
AUC: 0.944
CI: 0.927−0.961
PRAD
HEIH
AUC: 0.871
CI: 0.838−0.905
READ
HEIH
AUC: 0.910
CI: 0.879−0.940
THCA
HEIH
AUC: 0.803
CI: 0.773−0.833
UCEC
HEIH
AUC: 0.932
CI: 0.902−0.962
GBMLGG
HEIH
AUC: 0.923
CI: 0.910−0.936
GBM
HEIH
AUC: 0.918
CI: 0.891−0.946
LGG
HEIH
AUC: 0.925
CI: 0.912−0.938
OV
HEIH
AUC: 0.953
CI: 0.930−0.975
TGCT
HEIH
AUC: 0.968
CI: 0.950−0.986
THYM
HEIH
AUC: 0.843
CI: 0.809−0.878
UCS
HEIH
AUC: 0.990
CI: 0.979−1.000

Fig. S1-3

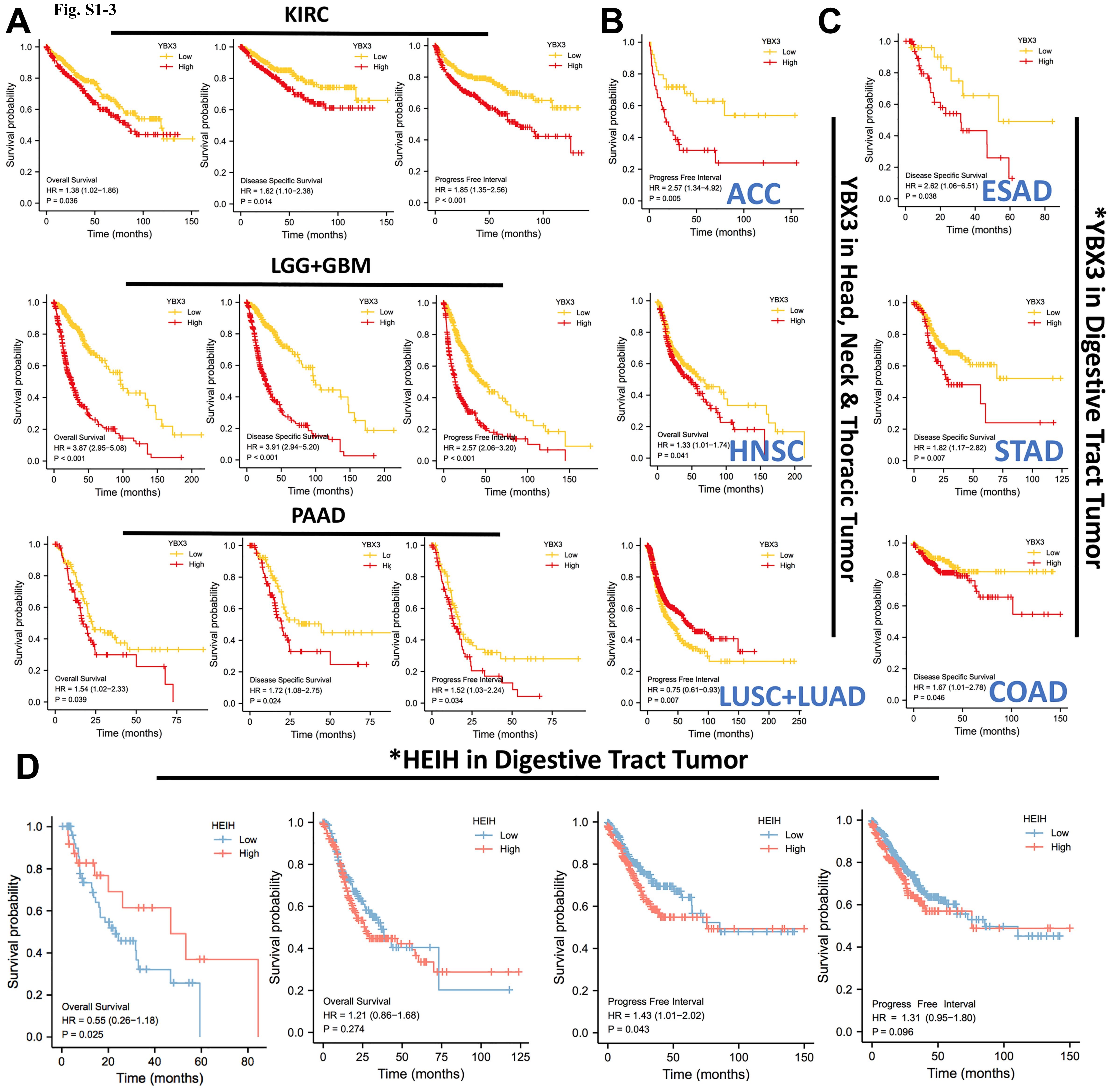

A
KIRC
LGG+GBM
PAAD
B
ACC
HNSC
LUSC+LUAD
C
ESAD
STAD
COAD
YBX3 in Head, Neck & Thoracic Tumor
*YBX3 in Digestive Tract Tumor
D
*HEIH in Digestive Tract Tumor
Overall Survival
HR = 1.38 (1.02−1.86)
P = 0.036
Disease Specific Survival
HR = 1.62 (1.10−2.38)
P = 0.014
Progress Free Interval
HR = 1.85 (1.35−2.56)
P < 0.001
Overall Survival
HR = 3.87 (2.95−5.08)
P < 0.001
Disease Specific Survival
HR = 3.91 (2.94−5.20)
P < 0.001
Progress Free Interval
HR = 2.57 (2.06−3.20)
P < 0.001
Overall Survival
HR = 1.54 (1.02−2.33)
P = 0.039
Disease Specific Survival
HR = 1.72 (1.08−2.75)
P = 0.024
Progress Free Interval
HR = 1.52 (1.03−2.24)
P = 0.034
Progress Free Interval
HR = 2.57 (1.34−4.92)
P = 0.005
Overall Survival
HR = 1.33 (1.01−1.74)
P = 0.041
Progress Free Interval
HR = 0.75 (0.61−0.93)
P = 0.007
Disease Specific Survival
HR = 2.62 (1.06−6.51)
P = 0.038
Disease Specific Survival
HR = 1.82 (1.17−2.82)
P = 0.007
Disease Specific Survival
HR = 1.67 (1.01−2.78)
P = 0.046
Overall Survival
HR = 0.55 (0.26−1.18)
P = 0.025
Overall Survival
HR = 1.21 (0.86−1.68)
P = 0.274
Progress Free Interval
HR = 1.43 (1.01−2.02)
P = 0.043
Progress Free Interval
HR = 1.31 (0.95−1.80)
P = 0.096
YBX3
HEIH
Low
High
Survival probability
Time (months)

Fig. S1-4

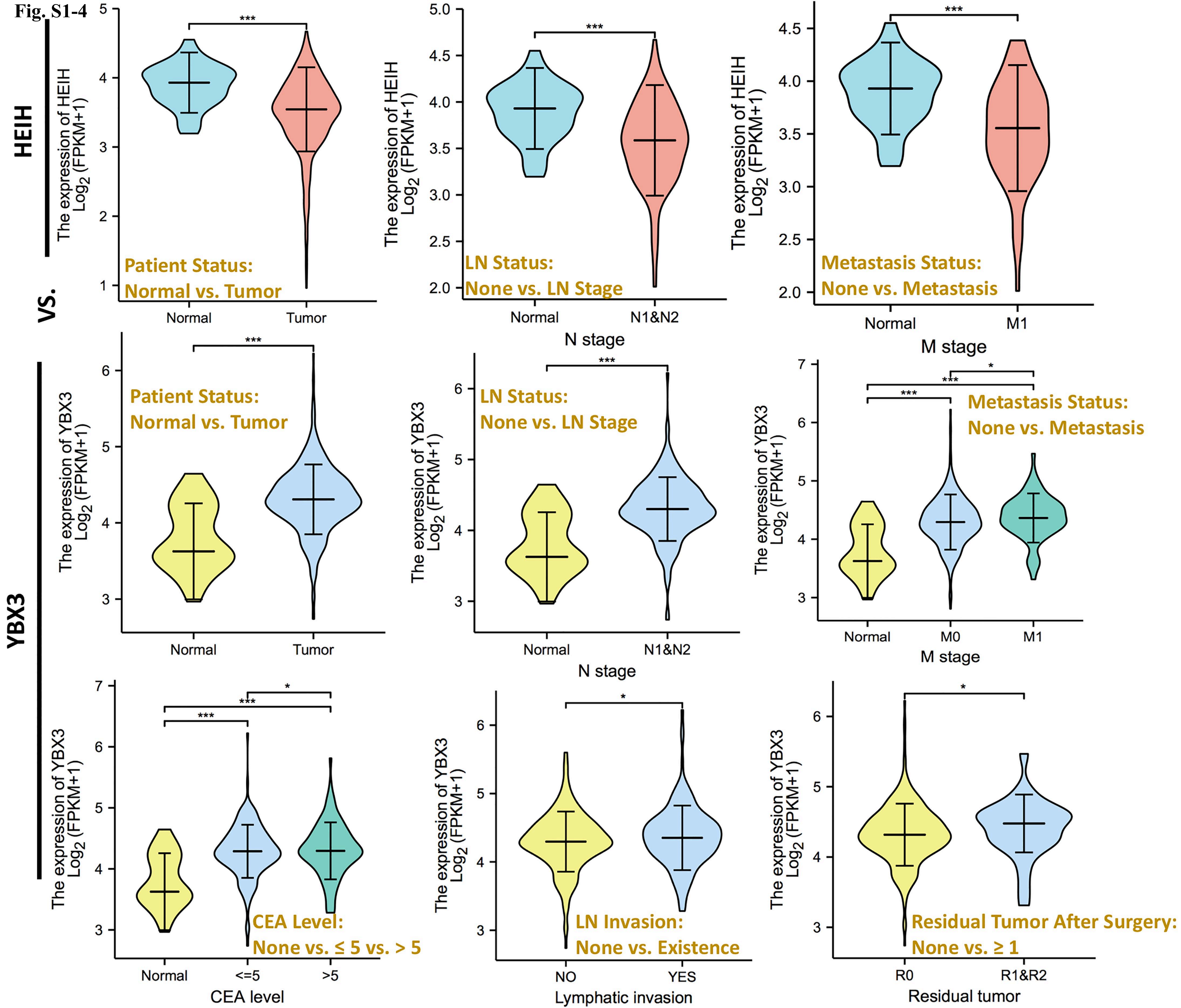
HEIH
VS.
YBX3
The expression of HEIH Log2 (FPKM+1)
Patient Status:
Normal vs. Tumor
Normal
Tumor
LN Status:
None vs. LN Stage
Normal
N1&N2
N stage
Metastasis Status:
None vs. Metastasis
Normal
M1
M stage
The expression of YBX3 Log2 (FPKM+1)
Patient Status:
Normal vs. Tumor
LN Status:
None vs. LN Stage
Metastasis Status:
None vs. Metastasis
Normal
M0
M1
M stage
CEA Level:
None vs. ≤ 5 vs. > 5
Normal
<=5
>5
CEA level
LN Invasion:
None vs. Existence
NO
YES
Lymphatic invasion
Residual Tumor After Surgery:
None vs. ≥ 1
R0
R1&R2
Residual tumor

Fig. S1-5

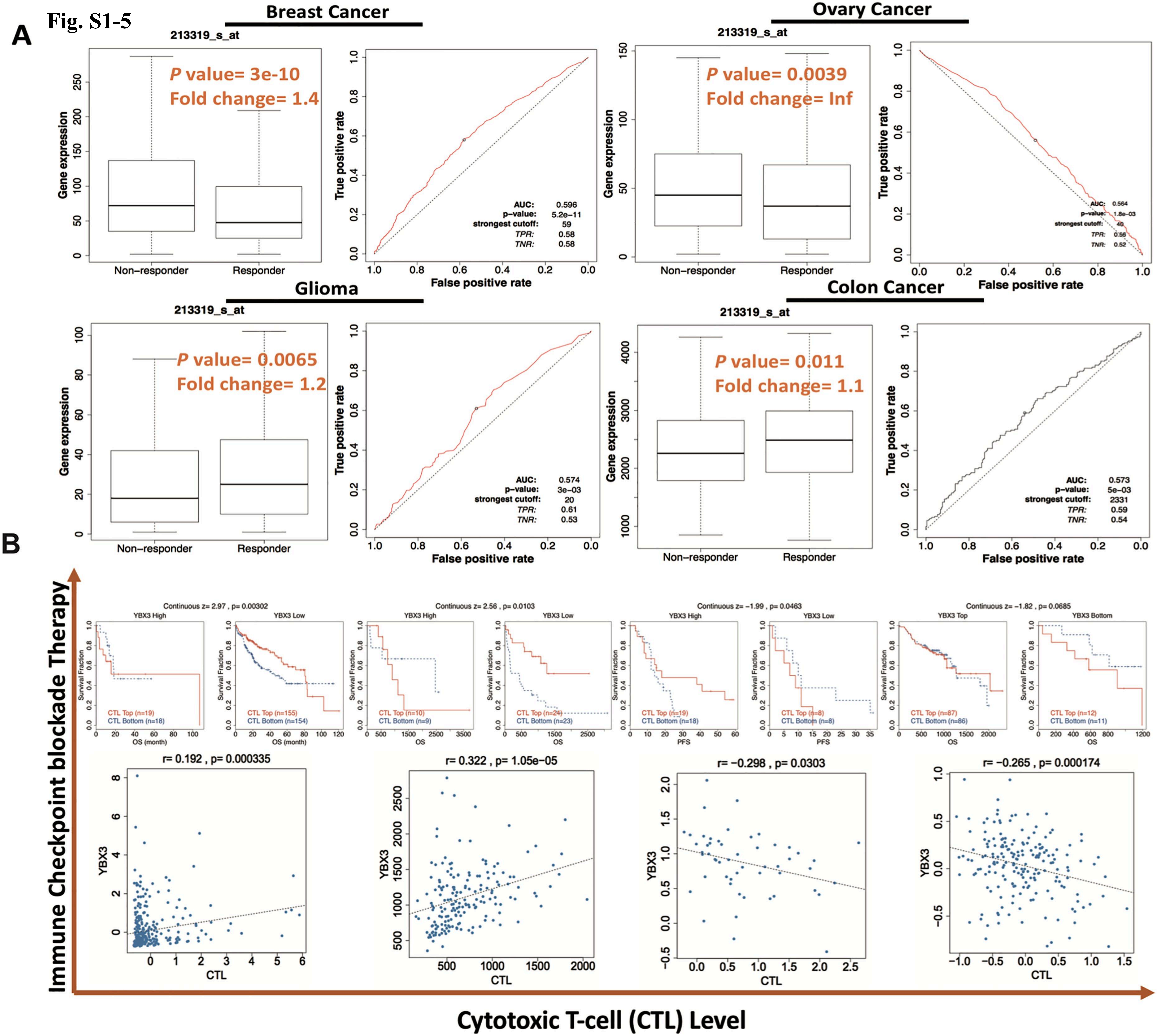
A
Breast Cancer
213319_s_at
P value= 3e-10
Fold change= 1.4
Gene expression
Non−responder
Responder
True positive rate
False positive rate
AUC: 0.596
p-value: 5.2e−11
strongest cutoff: 59
TPR: 0.58
TNR: 0.58
Ovary Cancer
213319_s_at
P value= 0.0039
Fold change= Inf
AUC: 0.564
p-value: 1.8e-03
strongest cutoff: 40
TPR: 0.56
TNR: 0.52
Glioma
213319_s_at
P value= 0.0065
Fold change= 1.2
AUC: 0.574
p-value: 3e-03
strongest cutoff: 20
TPR: 0.61
TNR: 0.53
Colon Cancer
213319_s_at
P value= 0.011
Fold change= 1.1
AUC: 0.573
p-value: 5e-03
strongest cutoff: 2331
TPR: 0.59
TNR: 0.54
B
Immune Checkpoint blockade Therapy
Continuous z= 2.97 , p= 0.00302
YBX3 High
YBX3 Low
CTL Top (n=19)
CTL Bottom (n=18)
CTL Top (n=155)
CTL Bottom (n=154)
OS (month)
Continuous z= 2.56 , p= 0.0103
CTL Top (n=10)
CTL Bottom (n=9)
CTL Top (n=24)
CTL Bottom (n=23)
OS
Continuous z= −1.99 , p= 0.0463
CTL Top (n=19)
CTL Bottom (n=18)
CTL Top (n=8)
CTL Bottom (n=8)
PFS
Continuous z= −1.82 , p= 0.0685
YBX3 Top
YBX3 Bottom
CTL Top (n=87)
CTL Bottom (n=86)
CTL Top (n=12)
CTL Bottom (n=11)
Survival Fraction
r= 0.192 , p= 0.000335
r= 0.322 , p= 1.05e−05
r= −0.298 , p= 0.0303
r= −0.265 , p= 0.000174
YBX3
CTL
Cytotoxic T-cell (CTL) Level

**Fig. S1-6**

# Flow Chart of Manuscript

① [Fig. 1] LncRNA-HEIH and YBX3 expression in various cancers (which finds that the trends of the expression of the 2 genes are quite opposite in the same cancer type, and here is the first evidence suggesting that the potential interaction between HEIH and YBX3)

② [Fig. S1-1] The YBX3 expression in different molecular and immune subtypes of various TCGA cancer types (which illustrates that there is relatively obvious difference of YBX3 expression among diverse subtypes of different TCGA cancer types, suggesting YBX3 is a potential immune biomarker)

③ [Fig. S1-2]The role of LncRNA-HEIH/YBX3 in the field of clinical diagnosis of different cancers (which proved the 2 points below: 1. Both LncRNA-HEIH and YBX3 have huge diagnostic potential in cancers. 2. Similar diagnostic ability indicating the two factors can form an integral oncogenic predicting system)

④ [Fig. S1-3 and 4]The role of HEIH and YBX3 in prognosis of digestive tract tumors (the results indicate that the two play just quite opposite roles in prognosis of digestive system cancers and their effects on prognosis are significant, which, from a clinical view, reconfirms that the HEIH and YBX3 can constitute an integral oncogenic system, especially in gastrointestinal cancers)

***Transition: from pancaner to specific caner, namely, from commonality to individuality***

⑤ [Fig. 2]The role of LncRNA-HEIH/YBX3 in crucial clinical characteristics of colon cancer (which further identified the oncogenic system composed by the two, and meanwhile, setting a stage for their potential effect on immunotherapy)

⑥ [Fig. 3-6]The DEGs of LncRNA-HEIH and YBX3 in colon cancer → constructions of hub genes networks → the distribution of their expression in immune cells via scRNA-seq and their inducing the immune infiltration as well as T-cell exclusion in colon cancer (which provides further evidence for immunotherapeutic target)

⑦ [Fig. S1-5] the effect of the oncogenic system on immune checkpoint blockade therapy in colon cancer (identifying its potential in ICB of cancer)

***Experimental Validation***

⑧[Fig. 7] Taking colon cancer as an example: proving that the interaction between LncRNA-HEIH and YBX3 can form a carcinogenic system in colon cancer from the aspect of experiment

Annotation of Border Color

* Green: The interaction of LncRNA-HEIH and YBX3 in pan-cancer (from plane-commonality).

* Red: The interaction of LncRNA-HEIH and YBX3 in digestive tract tumors (line-transition).

* Black: The interaction of LncRNA-HEIH and YBX3 in colon cancer (to point-individuality).
